# CRISPR-mediated genome editing in poplar issued by efficient transformation

**DOI:** 10.3389/fpls.2023.1159615

**Published:** 2023-04-17

**Authors:** Ali Movahedi, Hui Wei, Saeid Kadkhodaei, Weibo Sun, Qiang Zhuge, Liming Yang, Chen Xu

**Affiliations:** ^1^ College of Biology and the Environment, Nanjing Forestry University, Nanjing, China; ^2^ College of Arts and Sciences, Arlington International University, Wilmington, DE, United States; ^3^ Key Laboratory of Landscape Plant Genetics and Breeding, School of Life Sciences, Nantong University, Nantong, China; ^4^ Agricultural Biotechnology Research Institute of Iran, Isfahan Branch, Agricultural Research, Education and Extension Organization, Isfahan, Iran; ^5^ Nanjing Key Laboratory of Quality and Safety of Agricultural Product, Nanjing Xiaozhuang University, Nanjing, China

**Keywords:** CRISPR, efficient transformation, efficient HDR, DDT, poplar

## Abstract

**Background:**

CRISPR has been increasingly used for plant genetic improvements because of its high efficiency and precision. Recently, the authors have reported the possibility of homology-directed repair (HDR) using CRISPR/Cas9 through woody plants such as poplar. HDR often replaces nucleotides with one donor DNA template (DDT), including homologous sequences.

**Methods:**

CRISPR–Cas9 was recruited, and three variables, Agrobacteria inoculator concentration, pDDT/pgRNA ratio, and homologous arm length, were designed to integrate *nptII* and 2XCamV 35S into the *MKK2* promoter zone.

**Results:**

Here, we showed that recovered poplars on kanamycin-supplemented media exhibited enhanced expression of *MKK2* affected by the precise integration of 2XcamV 35S and *nptII*, improving biochemical and phenotypic properties. Our findings confirmed that *Agrobacterium* inoculator OD_600_ = 2.5, increased DDT numbers during cell division to 4:1 pDDT/pgRNA, and optimized homologous arms 700 bp caused efficient HDR and increased *MKK2* expression.

**Conclusion:**

Efficient transformations resulted from optimized variables, directly affecting the HDR efficiency through woody plants such as poplar.

## Introduction

Genetic improvements and RNA-guided genome editing of plants are currently possible using bacterial type II (*Streptococcus pyogenes*) clustered regularly interspaced short palindromic repeats (CRISPR) associated with a potential nuclear-localized endonuclease (Cas) and gRNA. In the CRISPR/Cas9 system, one chimeric gRNA molecule includes CRISPR RNA (crRNA) and trans-activating crRNA (tracrRNA), which drive Cas9 to cleave target sequences followed by at least 15-nucleotide (without mismatch at the 5′-end) spacer sequences complemented by target DNA. The Cas9 protein contains two DNA cleavage domains, HNH and RuvC, that cleave DNA to form a double-stranded break (DSB), typically 3 bp upstream of the protospacer adjacent motif (PAM) on the target DNA, especially 5′-NGG or 5′-NAG sequences. DNA system repairs include homology-directed repair (HDR) (Rouet et al., 1994; Bibikova et al., 2003) and non-homologous end-joining (NHEJ) (Bibikova et al., 2002).

HDR often replaces nucleotides with one donor DNA template, including homologous sequences, while NHEJ repairs damaged DNA by inserting or deleting (indel) nucleotides in DSB regions. Recently, researchers have indicated the abilities of stable transformation procedures using CRISPR/Cas9 (Di Fan et al., 2015; Howells et al., 2018). In addition, some plant genomes, such as wheat (Wang et al., 2014), *Arabidopsis thaliana* (Feng et al., 2013), *Nicotiana benthamiana* (Nekrasov et al., 2013), sorghum (Jiang et al., 2013), maize (Char et al., 2017), and poplar (Di Fan et al., 2015; Zhou et al., 2015), have been edited by the CRISPR–Cas9 system. Recently, the authors have shown that a poplar genome could be edited precisely by CRISPR–Cas9 under optimized conditions (Movahedi et al., 2022). In this published paper, the authors revealed how they applied the optimized delivery conditions (presented in the current paper) to overcome poplar genome editing through precise nucleotide replacements using CRISPR–Cas9. Moreover, the poplar genome has shown that CRISPR/Cas9 is a highly efficient tool for generating homozygous mutations and stable transformants through the first generation (Di Fan et al., 2015). It has been reported that virus-based replicons and particle bombardment could increase this translocation regarding the difficulty of donor DNA pattern (DDP) delivery to the cell nucleus (Gil-Humanes et al., 2017). The expanding number of cells containing DDTs at S/G2 cell division phases is essential for enhancing HDR efficiency (Yang et al., 2016). Attempts have been made to develop *Agrobacterium* transformation efficiency and to reveal that Agrobacteria inoculator OD directly affects transformation efficiency (Movahedi et al., 2014). However, still, there is no statement on enhancing the Agrobacteria method transfer to increase the efficiency of carrying DDP and the recovery of DSBs as HDR. *Mitogen-activated protein kinases* (*MAPKs*) are specific transferase kinase enzymes for threonine and serine amino acids. Recently, scientists have proved that *MAPKs* direct cellular responses to heat shock and osmotic stresses (Mohanta et al., 2015).

MAPK kinase (MAPKK or MKK) is a dual-specific protein kinase and needs phosphorylation to activate the MAPK pathway. In *Arabidopsis*, *MAPKK2* (*MKK2*) are transcriptional regulator genes stimulated by environmental stresses such as salinity and cold to promote plant resilience (Teige et al., 2004). *MKK4* and *MKK5*, which are more closely linked, share over 75% of their amino acids and seem to stimulate *MPK3* and *MPK6* in response to stress (Asai et al., 2002). *MKK5* displays upregulated expressions in the *MKK2* overexpression lines in response to stress regarding the activation of *MPK3* and *MPK6* (Asai et al., 2002). Upregulation of *MKK5* in the *MKK2* overexpression lines indicates the existence of a crosstalk between these pathways (Teige et al., 2004). Therefore, we focused on *MKK5* in this study as the downstream regulation of *MKK2* to investigate the *MKK2* expression.

Poplar is a fast-growing woody species planted extensively worldwide with significant ecological and economic value. Broad poplar genomic resources are available for studying functional genomics and performing model tree analyses. According to Polle et al. (2013), functional genomics studies in woody plants are problematic because of low-efficiency genetic transformations, few mutants, and a long growth period. We tried to integrate an exogenous promoter 2XcaMV 35S into the predicted promoter zone of the *MKK2* gene in the poplar genome for the first time. Moreover, we optimized a stable Agrobacteria transformation method and Cas9 nuclease to build DSBs at the specific sites on the *MKK2* gene. We recruited an HDR repair system to analyze the expression changes of the desired gene against salt stress and prove the role of the *MKK2* gene in poplar resistance.

## Materials and method

### MKK2 locus identification, target detection, and line generation

#### Identification and target detection

The *MAPKK* 2-like (*MKK2*) gene from *Populus euphratica* (XM_011018250.1) was searched to find similar sequences in *Populus trichocarpa* (*P. trichocarpa*) (XM_002324230.2) using the BLAST database of the National Center for Biotechnology Information (NCBI) (https://blast.ncbi.nlm.nih.gov/). Blasted sequences were aligned to reveal similarities between the *MKK2* gene from 14 plant species that exhibited highly conserved domains. The comparative analysis revealed that *MAPK* is a highly conserved gene family through plant species (Liu, 2012; Jiang and Chu, 2018). The phylogenetic tree was calculated to prove the similarities when comparing far genome distances with 1,000 replicates through bootstrap analysis (**Supplementary Figures 1**, **2**). The *MKK2* locus proximal promoter area was analyzed and confirmed by BDGP (Neural Network Promoter Prediction). Then, degenerate primers (F- and R-degeneracy; **Supplementary Table 1**) were designed using Geneious Prime^®^ 2021 (Biomatters Ltd., New Zealand) to isolate consensus DNA sequences from *P. trichocarpa*, which were then ligated into the pEASY-TA cloning vector and verified by Sanger sequencing (GenScript, Nanjing, China). Verified sequences were submitted to NCBI as *Populus trichocarpa* MAP kinase kinase family protein under accession number MG871465.

#### Line generation

To generate mutated *mkk2* lines or negative control (NCT), we applied Geneious Prime^®^ 2021 to analyze all the potential targets on the *MKK2* gene resulting in the detection of one conserved CRISPR target from both alleles of *MKK2* (chromosomes 6 and 18: NC_037290.1: 12,509,702–>12,513,458 and NC_037302.1: 4,954,681–>4,950,600), which was scored according to the on-target activity of 0.662 (Doench et al., 2014; Doench et al., 2016) and specificity score of 71.43% (Hsu et al., 2013) in blasting across the whole genome of *P. trichocarpa.* The detected CRISPR target was then applied to design oligos with *Bsa*I and ligate into the pGREB31 vector to construct pGREB-*mkk2* (*mkk2_F* and *mkk2_R*; **Supplementary Table 1**) (Xie et al., 2014).

To generate the *MKK2* overexpressed lines or positive control (PCT), we designed primers to PCR-amplified 2-kb genomic proximal promoter area from *P. trichocarpa* with *Bam*HI and *Kpn*I (Promo_F and Promo_R; **Supplementary Table 1**). The full-length CDS of *MKK2* was PCR-amplified from *P. trichocarpa* cDNA with *Bam*HI and *Pst*I to ligate with the proximal promoter and cloned into the pCAMBIA1300 vector to construct pCAM-*MKK2* (*MKK2*_full_F and *MKK2*_full_F; **Supplementary Table 1**).

To generate the *MKK2* edited lines (Ed), we analyzed the *MKK2* proximal promoter area on chromosome 18 to detect the potential CRISPR target, which was scored according to the on-target activity of 0.469 (Doench et al., 2014; Doench et al., 2016) and the specificity score of 83.33% (Hsu et al., 2013) in blasting across the whole genome of *P. trichocarpa* (Oligo-F and Oligo-R; **Supplementary Table 1**). A pair of oligos flanked by *Bsa*I adaptors were designed to anneal and phosphorylate for targeting. The synthesized oligos were ligated into digested pRGEB31 vectors by the *Bsa*I restriction enzyme to form pgRNA (Xie and Yang, 2013). All vectors were then transferred into *Escherichia coli* (DH5α) and propagated. Afterward, vectors were extracted using the plasmid midi kit (Qiagen, USA), and all ligated oligo sequences were confirmed by Sanger sequencing (GeneScript, Nanjing, China).

We transfected wild-type poplars to generate control lines (CT) using a pRGEB31 vector with no designed target gRNA scaffold (empty vector).

### Transformation

#### Control line transformation (NCT, PCT, and CT)

*Populus trichocarpa* seedlings were grown in a phytotron at 23°C ± 2°C under a 16/8 light/dark period. All tissue culture and transformation techniques were performed according to a recently published paper (Movahedi et al., 2015). The plasmids pGREB-*mkk2* for NCT, pCAM-*MKK2* for PCT, and empty vector for CT were then transferred into *Agrobacterium tumefaciens.* The *A. tumefaciens-*triggered explants were co-cultivated in a dark room for 2 days. Afterward, explants were cultured into a selection of semi-solid woody plant medium (WPM) containing 0.05 mg/L of indole-3-butyric acid (IBA), 0.004 mg/L of thidiazuron (TDZ), 400 mg/L of cefotaxime, 8 mg/L of hygromycin, and 0.8% (w/v) agar. After 4 weeks, the regenerated buds were subcultured in media with 0.05 mg/L of IBA, 0.001 mg/L of TDZ, 200 mg/L of cefotaxime, 8 mg/L of hygromycin, and 0.8% (w/v) agar, to shoot. The 8-week-old prolonged shoots were then introduced into the media with 100 mg/L of cefotaxime and 8 mg/L of hygromycin and allowed to root. The rooted explants were then assumed to be successfully transformed. Approximately 80 ± 10 independent biological replicates were generated for each NCT, PCT, and CT line.

#### Edited line transformation

To generate Ed lines, *P. trichocarpa* seedlings were used as the template for the above transformation techniques. Three Agrobacteria concentrations (OD_600_ = 0.5, 1.5, and 2.5), three ratios of pDDT (plasmid including donor DNA template) and pgRNA (plasmid including target scaffold) (1:4, 1:1, and 4:1), and four different lengths of homologous arms to generate pDDT (500, 600, 700, and 800 bp) were selected as the variables in this study and classified into nine groups (**Supplementary Table 2**). Approximately 80 ± 10 independent biological replicates were generated for each variable (**Supplementary Table 2**). The 8-week-old prolonged shoots were then transferred into selective media with 200 mg/L of cefotaxime and 25 mg/L of kanamycin and allowed to root. Moreover, the CT lines were assumed as 0 bp of homologous arms and applied separately for each group as the control to subculture into selective media with 200 mg/L of cefotaxime and 25 mg/L of kanamycin. All groups were then analyzed to calculate and compare the transformation efficiency (**Supplementary Tables 2**, **3**).

### Verification of negative and positive lines and survival rates

To verify the NCT lines, we used *mkk2* mutants as a template resource for *Agrobacterium* transformation and then transferred pCAM-*MKK2* into *mkk2* explants regarding the above methods leading to reactive *MKK2* expression and generating mutant-overexpressions (M-OEs). Approximately 80 ± 5 M-OEs were approved for hygromycin screening. Eight-week-old recovered explants from the NCT, PCT, M-OEs, and CT lines were transferred to MS media supplemented with 0, 25, and 50 mM of NaCl (20 independent biological replicates for each NaCl concentration) and allowed to grow for 3 weeks. After that, we synthesized cDNAs from all transformed NCT, PCT, M-OEs, and CT lines and used the Applied Biosystems real-time PCR (Applied Biosystems, USA) to analyze the expression of the *MKK2* gene (*MKK2*_full_F and *MKK2*_full_R; **Supplementary Table 1**) via NaCl treatments for comparison and verification (three technical repeats for each biological replicate).

In addition, we allowed the NaCl-treated poplars to recover for 1 week by regulatory watering. Then, all 4-week-old transformed explants were investigated for survival rates [(Survived living explants: Total seedling transformed) × 100].

### *MKK5* expression analysis

To verify the NCT lines, we further analyzed the *MKK5* (chromosome 8; NC_037292.1) expression that was downstream-regulated by the *MKK2* gene using designed primers by Geneious Prime^®^ (*MKK5*_F and *MKK5*_R; **Supplementary Table 1**) to isolate 1,071 bp from *MKK5* CDS. All transformed explants were introduced to different NaCl treatments. Then, the synthesized cDNAs from all transformed NCT, PCT, M-OEs, and CT lines were used for real-time PCR (three technical repeats for each biological replicate).

### Plasmid design and cassettes

The 2XCaMV 35S promoter was isolated from the pCre vector (DQ645631) using 2X-F-pCre and 2X-R-pCre primers (**Supplementary Table 1**). The 2X-F primer was used to add 30 bp homology sequences from the 5′ region of *nptII*, and the 2X-R primer was used to add 40 bp homology sequences from the 3′ region of the upstream homologous (UH) arm (**Supplementary Table 1**; **Supplementary Figure 3A**). The neomycin phosphotransferase (*nptII*) CDS was selected as a resistant gene against kanamycin and isolated from the PBI121 (AF485783) plasmid using *nptII*-F-PBI and *nptII*-R-PBI primers (**Supplementary Table 1**). The *nptII*-F primer was then used to add 40 bp homology sequences from the 5′ region of the downstream homologous (DH) arm (**Supplementary Figure 3B**). The PCR products of the overhung 2XCaMV 35S and *nptII* were then ligated using PCR to form a pre-cassette (**Supplementary Figure 3C**). To ligate products using PCR, we need a 30–40-bp overhang on one of the PCR products complementary to the end of the adjacent PCR product (using initial primers). It is necessary to allow the PCR products to anneal at a higher temperature to avoid non-specific hybridization through the long PCR products, which are non-complementary, and then fill up the PCR tubes with the final primers to make a double-stranded product. Therefore, three cycles of annealing (68°C) and extension (74°C) were arranged, and the desired primers were supplemented to the distal ends of the fragments using a regular PCR. The primers Back-F and Back-R (**Supplementary Table 1**) were then applied to form the cassette backbone and were confirmed by sequencing (**Supplementary Figure 3D**). To optimize the length of the homologous arms, nucleotides of different lengths from both sides of the selected target (500, 600, 700, and 800 bp) were used to construct cassettes including different homologous arms. The final constructed cassettes were cloned into the pGEM-T easy vector (Promega, New Zealand) for verification by sequencing. Briefly, the DH homologous arm was isolated from genomic DNA using 500-DH-F and 500-DH-R (**Supplementary Table 1**). To add the T-DNA left border, we used the previous PCR product as the template and amplified that again using T500-DH-F and 500-DH-R primers (**Supplementary Table 1**; **Supplementary Figure 4A**). The UH homologous arm also was isolated from genomic DNA using 500-UH-F and 500-UH-R (**Supplementary Table 1**; **Supplementary Figure 4B**). As described, two isolated homologous arms and the cassette backbone were then ligated by PCR to form the cassette (**Supplementary Figure 4C**). All lengths of the homologous arms were made with the same method as described and used appropriate primers (**Supplementary Table 1**). The verified cassettes were then ligated into the pRGEB31 plasmid using the restriction cloning technique to create pDDT (**Supplementary Figure 4D**).

### Genomic DNA and total RNA extraction and cDNA synthesis

Genomic DNA was extracted from CT, NCT, and edited events grown on kanamycin using the CTAB method. Briefly, 80-100 µg of young leaves were quickly ground in liquid nitrogen, and 200 µl of 65°C preheated CTAB was added to the samples, followed by five inversions. Then, the samples were incubated at 65°C for 30 min, supplanted by adding 200 µl of chloroform, incubated at room temperature (RT) for 5 min, and centrifuged at 16,000*g* for 10 min at 4°C. The supernatant was then shifted to a new tube, mixed with 300 µl of isopropanol, and incubated on ice for 30 min. The precipitated genomic DNA was then separated via 16,000*g* centrifugation for 20 min at 4°C. The pellet was then washed twice with 500 µl of 70% ethanol. The semidried pellet was dissolved in 100 µl of double-distilled water and stored at 4°C. The combination of the obtained genomic DNA was measured using the BioDrop spectrophotometer at 1,300 ng/µl. Total RNA (100 ng/ml) was also extracted from the young transformant leaves grown on kanamycin and WT applying TRIzol. According to the manufacturer, we then adopted reverse transcription using total RNA and oligo-dT primers to synthesize the first cDNA strand (PrimeScript One-Step RT-PCR Kit Ver.2, Takara Biotechnology, Dalian, China).

### Integration confirmation and off-target activity

To verify *nptII* integration and off-target activity incidents mediated by CRISPR genome editing, we used extracted genomic DNA from CT, NCT, and edited events (Ed_23_1, Ed_31_1, Ed_34_1, Ed_35_1, Ed_35_2, Ed_35_3, and Ed_36_1) to amplify 1,457 bp of a PCR fragment from *nptII* as the reverse primer and nucleotides out of the 3′ homologous arm (downstream homologous) on the second intron as the forward primer (5,552 F, 7,008 R; **Supplementary Table 1**). ImageJ ver.2 was applied to measure on- and off-target intensities.

### TaqMan real-time PCR for HDR efficiency

To test the proper direction of combining exogenous *nptII*, we used extracted genomic DNA as the template to run the TaqMan assay applying dye labels such as FAM and VIC using Applied Biosystems real-time PCR (Applied Biosystems, USA) (Movahedi et al., 2022). The Ed_23_1, Ed_31_1, Ed_34_1, Ed_35_1, Ed_35_2, Ed_35_3, and Ed_36_1 recovered events were used in this method. Thus, we designed primers to probe three 90-bp fragments: FAM1, 2, and 3 (**Supplementary Table 1**). These primers were planned to probe 52 bp of downstream homologous and 38 bp from the 3′ end of *nptII* (FAM1), 44 bp of *nptII* and 46 bp of 2XCamV 35 (FAM2), and 37 bp of 2XCamV 35 and 53 bp of upstream homologous (FAM3). In addition, the primers probed one 106 bp fragment VIC on the *β-actin* gene as the reference with a stable copy number (**Supplementary Table 1**, 1117-F and 1222-R). All samples were analyzed in quadruplicates. TaqMan uses a fluorogenic probe to bind 20 single-stranded nucleotides only between forward and reverse primers. Therefore, only completed strands could be assigned by this probe and generate the fluorescent signals in TaqMan real-time PCR. The signals detected from each FAM were compared to VIC signals to calculate ΔΔC_t_ and HDR efficiency (%).

### Polymorphisms

NHEJ is characterized by introducing irregular small indels into the targeted site. However, regardless of this mutagenic potential and its propensity for error, NHEJ plays an active role in repairing genome integrity and suppressing chromosomal translocations and in the bulk of repair events in the genome (Ceccaldi et al., 2016).

We analyzed the variant genotypes within UH, DH, and knocked-in fragments from recovered events resulting from CRISPR activity to test the effect of HDR promotions resulting from optimized transformation on polymorphisms. Five polymorphism varieties were detected, including deletions, insertions, SNP transitions (A to C or G to T and vice versa), SNP transversions (purines to pyrimidines or vice versa), and substitutions. All genomic DNA extracted from the recovered events was then isolated by the designed primers (Seq_F and Seq_R; **Supplementary Table 1**), cloned into a pEASY-TA cloning vector, and sequenced (GeneScript, Nanjing, China) to detect polymorphisms that occur using Geneious Prime 2022.

### Phenotypic properties

It has been shown that *MAPK* genes direct cellular responses against abiotic stresses such as salinity (Teige et al., 2004; Mohanta et al., 2015; Sun et al., 2015) and regulate cell growth and death, differentiation, and the cell cycle (Jonak, 2002; Yang et al., 2017). Regarding these findings, we measured chlorophyll and carotenoid contents and root length before and after salt stress to prove the proper HDR and evaluate the effect of 2XCaMV 35S on *MKK2* expression among CT, NCT, and recovered events.

### Statistical analysis

All data were analyzed using one-way ANOVA with Tukey’s *post-hoc* comparisons calculated by GraphPad Prism 9.3 (GraphPad Software, LLC). Differences were analyzed when the confidence intervals presented no overlap of the mean values with an error value of 0.05.

## Results

### Mutant lines were confirmed via salt treatments

**Figure 1:**
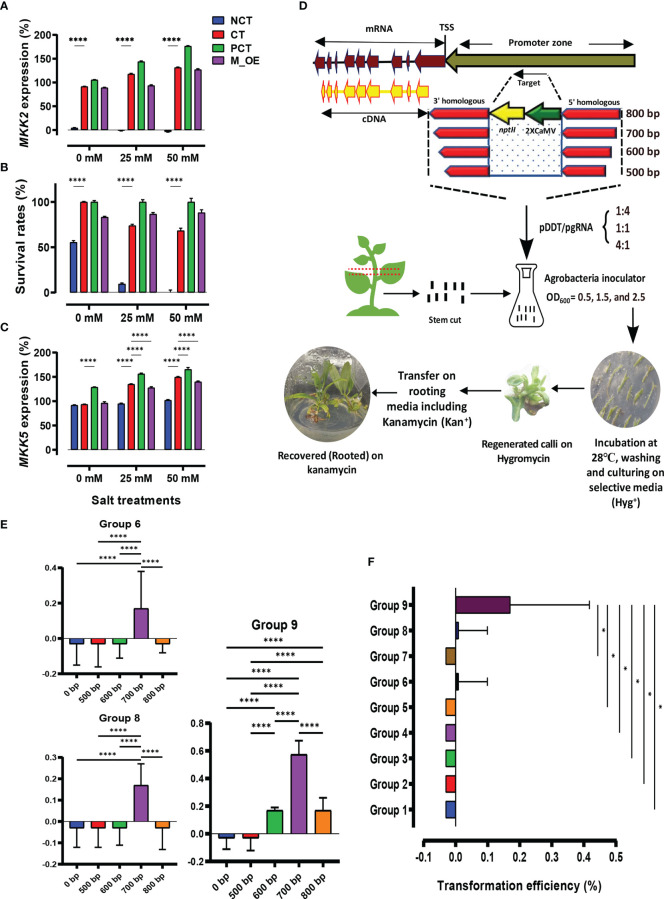
Graphical summary shows the designed experiments for transformation assessment and homology-directed repair (HDR) efficiency. **(A–C)** Control (CT) and negative control (NCT) verification among *MKK2*, survival treatments, and *MKK5* expression. **(D)** Different homologous arm lengths, Agrobacteria inoculators, and the proportion of pDDT/pgRNA caused efficient transformation screened by media-supplemented kanamycin. Transformed explants were regenerated on hygromycin-selective media and rooted in kanamycin-selective media. **(E)** Mean comparison of recovered events influenced by different homologous arms. **(F)** Transformation efficiency (%) evaluation through designed groups; error bars represent SE; asterisks represent *p*-values as * ≤0.05, and **** ≤0.0001.

The *mkk2* mutant lines (NCT) were investigated for lacking *MKK2* expression (%) through NaCl treatments and in comparison with the CT lines (**Figure 1A**). While the NCT lines revealed no *MKK2* expression, the CT and PCT lines indicated incremental expressions via increasing salt treatments. The M_OE lines only revealed increased *MKK2* expression (%) in the 50-mM NaCl concentration. We analyzed survival rates (%) to further confirm the proper *mkk2* mutants compared with the CT lines (**Figure 1B**). While the CT, PCT, and M_OE lines revealed acceptable survival rates (%) among various NaCl treatments, the NCT lines were negatively affected by the high salt concentration of 50 mM. It has been proved that the expressed *MKK2* activated by cold and salt stresses upregulates the *MKK5* expressions (Teige et al., 2004).

Regarding these findings, the lack of *MKK2* expression causes the downregulation of *MKK5* under salt stress. We analyzed the *MKK5* expressions affected by *MKK2* silencing to further verify the proper *mkk2* mutant lines compared with the CT lines (**Figure 1C**). The results revealed significant incremental *MKK5* expression in the CT lines compared with the NCT lines under 25 and 50 mM of NaCl concentrations. The M_OE and PCT lines also revealed acceptable increased *MKK5* expression under 25 and 50 mM of NaCl concentrations.

### The efficient transformation resulted in the generation of recovered events

A schematic diagram of the designed experiments showed the target site, which was in the promoter zone of the *MKK2* locus (**Figure 1D**). This target was replaced by different homologous arms associated with the *nptII* CDS and 2XCamV 35S promoter among different ratios of pDDT/pgRNA (**Figure 1D**). The arranged groups, including different variables (**Supplementary Table 2**), revealed various regenerated edited events (Eds) on the screen media supplemented by hygromycin. Most regenerated Eds were observed through groups 6, 8, and 9 with Agrobacteria inoculators OD_600_ = 2.5, 1.5, and 2.5, respectively. In addition, groups 8 and 9 exhibited more regenerated Eds than group 6, with a pDDT/pgRNA ratio of 4:1 (**Supplementary Table 2**; **Supplementary Figure 5**). These results revealed recovered events (rooted in supplemented media with kanamycin) only in groups 6, 8, and 9 (**Supplementary Figure 5**). In addition, only recovered Eds with 700 bp homologous arms (group 9) showed significant differences compared with the CT lines (**Supplementary Figure 5**). The comparisons of all the lengths of homologous arms through groups 6, 8, and 9 revealed that Eds with 700 bp homologous arms were significantly more than the other events with 0 (CT), 500, 600, and 800 bp (**Figure 1E**). Concerning all variables and Eds, group 9 revealed significantly more transformation efficiency (%) (recovered Eds) than the other groups (**Figure 1F**; **Supplementary Table 3**). In total, the results showed that 700 bp of homologous arms were associated with high concentrations of Agrobacteria inoculation (OD_600_ = 2.5) and pDDT/pgRNA ratio of 4:1, significantly improving transformation efficiency (%) to recover edited events.

### HDR was confirmed by recombinant mRNA

**Figure 2:**
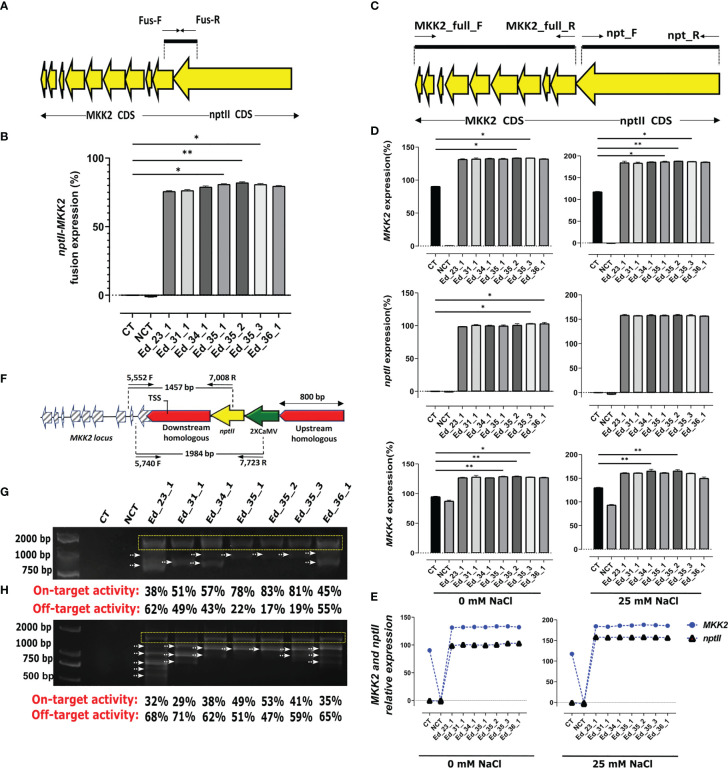
Recovered edited events exhibited nptII–MKK2 fusion and proper exogenous and exopromoter integration. **(A)** Schematic of *nptII*–*MKK2* mRNA fusion. **(B)** nptII and MKK2 fusion mRNA revealed more expression via recovered events, especially Ed_35_1, Ed_35_2, and Ed_35_3, compared with the CT line. **(C)** Schematic of complete CDS of nptII and *MKK2* through proper HDR. **(D)** The recovered edited events showed *MKK2*, *nptII*, and the downstream *MKK5* more expressions than the CT line under 0 and 25 mM of NaCl treatments. **(E)** *nptII* and *MKK2* exhibited relative expressions under 0 and 25 mM of NaCl treatments. **(F)** Schematic of properly integrating *nptII* and 2XCaMV 35S into *MKK2* promoter zone and overexpressed *MKK2* driven by 2XCaMV 35S. **(G)** PCR-amplified verification of *nptII* proper integration and on- and off-target evaluation. **(H)** PCR-amplified validation of 2XCaMV 35S proper integration and on- and off-target evaluation. Error bars represent SE; asterisks represent *p*-values as *≤ 0.05 and ** ≤0.01.

The supposed recombinant mRNA resulting from successful HDR was assessed using *MKK2* and *nptII* fusion (**Figure 2A**). Regarding this hypothesis, *MKK2* and *nptII* mRNAs should be fused to each other and tracked by one shared probe (**Figure 2A**). Designed primers (FUS_F and FUS_R; **Supplementary Table 1**) were applied to detect the expression of this fusion by real-time PCR from synthesized cDNA. All recovered Eds revealed fusion expression compared with the CT lines (**Figure 2B**). Regarding these results, Ed_35_1, Ed_35_2, and Ed_35_3 exhibited significant fusion expressions compared with the other Eds. To further prove proper HDR through recovered Eds, the complete CDS of *MKK2* and *nptII* was real-time PCR-amplified using designed primers (MKK2_full_F and MKK2_full_R and nptII_F and nptII_R; **Supplementary Table 1**) to evaluate their expressions (**Figure 2C**). All the recovered Eds showed *MKK2* expression (%) more than the CT lines affected by the precise integration of the 2XCaMV 35S promoter under 0 mM of NaCl treatment (**Figure 2D**). Ed_35_2 and Ed_35_3 exhibited significant expressions compared with the CT lines. Increasing the NaCl concentration to 25 mM increased *MKK2* expression, in which Ed_35_1, Ed_35_2, and Ed_35_3 exhibited significant enhanced *MKK2* expression against the CT lines (**Figure 2D**). Moreover, the *nptII* expression analyses in 0 and 25 mM of NaCl proved its proper expression in recovered Eds compared with the CT lines with no expression (**Figure 2D**). The downstream *MKK5* regulated by *MKK2* expression was further analyzed under 0 and 25 mM of NaCl treatment (**Figure 2D**). The results revealed significant *MKK5* expression in Ed_35_1, Ed_35_2, and Ed_35_3 compared with the CT lines affected by more *MKK2* expression by integrating 2XCamV 35S under 0 mM of NaCl concentration. Increasing salt stress to 25 mM resulted in an enhanced *MKK5* expression through edited events compared with the CT lines. NCT lines revealed decreased *MKK5* expression through increasing NaCl concentrations, which was influenced by the lack of *MKK2* expression in *mkk2* mutant lines. Concerning the *MKK2* and *nptII* fusion mRNA, we evaluated the relative expressions among the recovered Eds and under 0 and 25 mM of salt stress. We found constant relative expressions between endogenous *MKK2* and integrated exogenous *nptII*, resulting in the exact HDR happening through the recovered Eds (**Figure 2E**).

### Recombinant genomic DNA has confirmed on-target activity

Specific primers were designed from the *nptII* and *MKK2* locus only to amplify the 1,457-bp fragment from successful HDR in recovered edited events (**Figure 2F**). To evaluate the recombinant genome, we used extracted genomic DNA from all recovered events to amplify PCR (5,552F and 7,008R; **Supplementary Table 1**) (**Figure 2F**). The recovered Eds revealed exactly 1,457-bp bonds, resulting in the precise *nptII* and *MKK2* locus integrations. Furthermore, several unexpected bonds were attended as off-target happenings. The specific and unspecific bonds were then analyzed to measure their intensities and determine on- and off-target activities through the recovered Eds (**Figure 2G**; **Supplementary Figure 6A**). To further verify the precise integration of 2XCamV 35S with the *MKK2* locus, specifically designed primers (5,740F and 7,723R; **Supplementary Table 1**) were applied to PCR amplify several nucleotides from 2XCamV 35S to intron 2 of the *MKK2* locus (**Figure 2F**). All the recovered Eds revealed 1,984-bp-specific bonds (**Figure 2H**; **Supplementary Figure 6B**). In addition, Ed_35_1, Ed_35_2, and Ed_35_3 showed lesser off-target activities but more on-target activities than the other recovered Eds.

### TaqMan real-time PCR revealed efficient HDR

**Figure 3:**
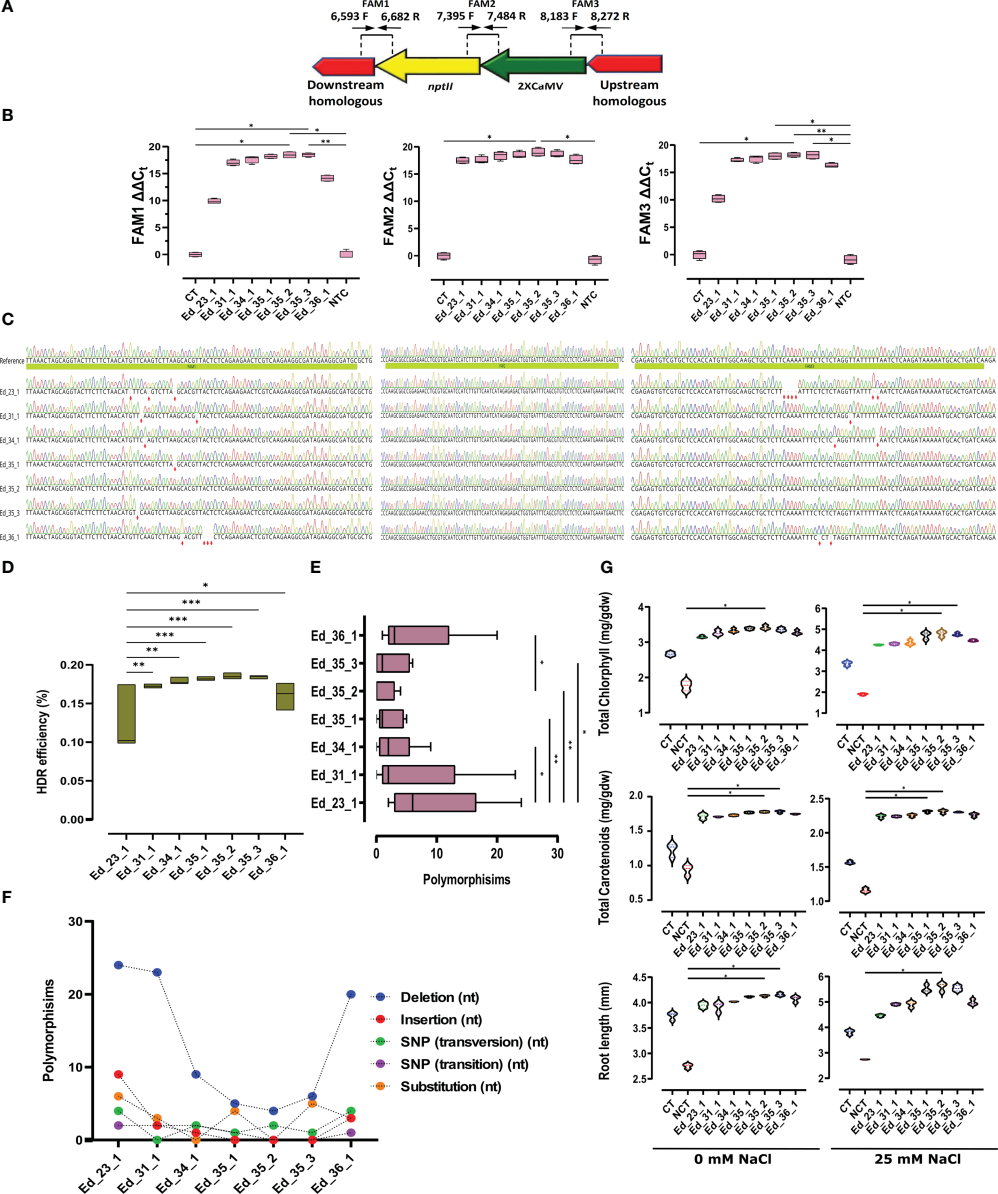
TaqMan real-time PCR exhibited efficient HDR through efficient transformant events. **(A)** Schematic FAM design to evaluate HDR efficiency. **(B)** Ed_35_1, Ed_35_2, and Ed_35_3 exhibited more FAM signals than the CT and NCT lines. Especially Ed_35_2 revealed significantly more expression signals within FAM1, 2, and 3 than the recovered edited events. **(C)** Sanger sequencing to verify results achieved by FAM signals. **(D)** HDR efficiency (%) evaluation calculated from the achieved FAM signals. Ed_35_1, Ed_35_2, and Ed_35_3 revealed more HDR efficiency. **(E)** Mean comparison of polymorphisms that happened through recovered edited events. **(F)** Polymorphisms exhibited more insertions and deletions among Eds except for Ed_35_1, Ed_35_2, and Ed_35_3 events. **(G)** Total chlorophyll and carotenoids calculated from the recovered edited events exhibited more through Ed_35_1, Ed_35_2, and Ed_35_3 than the other Eds under 0 and 25 mM of NaCl treatments. In addition, root length measurement confirmed more growth among Ed_35_1, Ed_35_2, and Ed_35_3 than the other Eds under 0 and 25 mM of NaCl treatments. Error bars represent SE; asterisks represent *p*-values as * ≤0.05, ** ≤0.01, and *** ≤0.001.

We applied FAM1, 2, and 3 to evaluate HDR efficiency (**Figure 3A**). FAM1 was primed (6,593F and 6,682R; **Supplementary Table 1**) to estimate HDR efficiency through the downstream homologous arm and *nptII*. FAM1 ΔΔC_t_ analyses revealed significant homology nucleotides in Ed_35_2 and Ed_35_3 compared with the CT and NCT lines (**Figure 3B**). Sanger sequencing among FAM1 further exhibited several mutant nucleotides within Ed_23_1, Ed_34_1, Ed_35_1, Ed_35_3, and Ed_36_1, except Ed_35_2 with no mutant nucleotides (**Figure 3C**). To estimate HDR efficiency between *nptII* and 2XCamV 35S, FAM2 was designed to prime (7,395F and 7,484; **Supplementary Table 1**). FAM2 ΔΔC_t_ analyses revealed homology nucleotides, resulting in sufficient FAM2 signals from all the recovered Eds (**Figure 3B**). Only Ed_35_2 showed significantly more signals compared with the CT and NCT lines. In addition, Sanger sequencing confirmed FAM2-sufficient homology nucleotides with no mutation (**Figure 3C**). To evaluate the upstream homologous arm and 2XCamV 35S homology nucleotides, FAM3 was primed (8,183F and 8,272R; **Supplementary Table 1**). While FAM3 ΔΔC_t_ analyses exhibited significant HDR efficiency through Ed_35_2 compared with the CT and NCT lines, Ed_35_1 and Ed_35_3 exhibited significantly more FAM3 signals than the NCT lines only (**Figure 3B**). While Sanger sequencing further confirmed FAM3 ΔΔC_t_ to show several mutant nucleotides through Ed_23_1, Ed_31_1, Ed_34_1, and Ed_36_1, Ed_35_1, Ed_35_2, and Ed_35_3 exhibited no mutant nucleotides (**Figure 3C**). HDR efficiency (%) was then evaluated by calculating all achieved FAM signals from the recovered Eds except for the CT and NCT lines with no HDR efficiency (**Figure 3D**; **Supplementary Table 4**). While Ed_31_1, Ed_34_1, and Ed_36_1 exhibited acceptable HDR efficiency compared with Ed_23_1 with the lowest efficient HDR, Ed_35_1, Ed_35_2, and Ed_35_3 exhibited more significant efficient HDR (**Figure 3D**).

### Efficient transformed edited events revealed minor polymorphisms

Total polymorphism analyses exhibited significantly lesser polymorphisms through the Ed_35_2 event than Ed_23_1 and Ed_36_1 with the most polymorphisms (**Figure 3E**; **Supplementary Table 5**). Moreover, Ed_35_1 and Ed_35_2 exhibited lower polymorphisms than Ed_23_1, Ed_31_1, Ed_34_1, and Ed_36_1 (**Figure 3E**). More detailed analyses revealed that maximum deletions occurred in Ed_23_1 genome editing, and minimum deletions happened in Ed_35_1, Ed_35_2, and Ed_35_3 (**Figure 3F**; **Supplementary Figure 7**). Ed_35_1, Ed_35_2, and Ed_35_3 revealed lower insertions through HDR happenings, while Ed_23_1, Ed_31_1, and Ed_36_1 exhibited higher insertion nucleotides (**Supplementary Figure 7**; **Supplementary Table 6**).

### Biochemical and phenotypic assessments proved efficient HDR through efficient transformants

Since *MKK2* is a positive regulator of poplar defense against environmental stresses, it is supposed that targeted *MKK2* over NHEJ may decrease plant resistance to salinity. Targeted *MKK2* over the HDR system to insert the exogenous 2XCaMV 35S promoter produced overexpressed *MKK2* (**Figure 2D**) compared with the CT and NCT lines and may promote resistance to salt stress. Regarding the direct positive effect of *MKK2* expression on phytohormones such as chlorophyll and carotenoids (Mao et al., 2017) and on plant growth and development (Nishihama et al., 1995), we analyzed all the recovered Eds for the contents of these phytohormones and further root development under salt treatments.

Total chlorophyll was detected in higher contents through all the recovered Eds than in the CT and NCT lines under 0 mM of NaCl concentration (**Figure 3G**). NaCl (25 mM) increased chlorophyll contents affected by overexpressed *MKK2* through 2XCamV 35S integration among the recovered Eds, but Ed_35_2 and Ed_35_3 exhibited significantly more contents than the NCT line (**Figure 3G**). To confirm the effect of enhanced *MKK2* expression resulting from the precise integration of HDR, we further analyzed carotenoid contents and found similar results of chlorophyll under 0 and 25 mM of salt stress (**Figure 3G**). The higher 25-mM NaCl concentration caused carotenoids to increase slightly in the CT and NCT lines but significantly increased carotenoids among Ed_35_1 and Ed_35_2. Finally, all the recovered Eds exhibited enhanced growth in root lengths under 0 mM of NaCl treatment. A high NaCl concentration of 25 mM revealed an acceptable enhancement in root length in Ed_35_1 and Ed_35_3 but caused significantly more root growth in Ed_35_2 compared with the NCT line (**Figure 3G**).

## Discussion

MPK pathways are involved in different physiological reactions and are conserved genetically in eukaryotes (Davis, 2000). Activating MPK pathways in response to stressors promotes plant stress tolerance (Sinha et al., 2011). In reaction to stress, *MKK4* and *MKK5* overexpression has been shown to promote *MPK3* and *MPK6* expression (Asai et al., 2002). MPK3 and MPK6 effectively phosphorylated IAA15 at the Ser-2 and Thr-28 residues (Kim et al., 2022), decreasing lateral root growth due to increased IAA15 accumulation, leading to inhibit the formation of lateral roots in response to drought. Moreover, increased *MKK5* expression caused by *MKK2* overexpression has been shown to be effective in triggering *MPK3* and *MPK6* in response to stresses (Asai et al., 2002), resulting in the significance of *MKK2* as a transcriptional regulator gene that makes plants more resistant to salt and cold (Teige et al., 2004).

CRISPR–Cas9 has been used for biotechnology, functional biology, and genetic medicine. *Agrobacterium rhizogenes* and *A. tumefaciens* have been used to transfer CRISPR-gRNA sites into plant cells (Jia and Wang, 2014; Di Fan et al., 2015). This research revealed that increasing OD_600_ to 2.5 improved HDR efficiency, while groups 6 and 9 revealed recovered events. In addition, OD_600_ = 0.5 exhibited no recovered events regardless of the pDDT/pgRNA ratio. Increasing OD_600_ to 1.5 caused recovery events within group 8. The other variable was the number of DDTs in the S/G2 cell division phases. According to Yang et al. (2016), the number of DDTs is critical in enhancing HDR efficiency. Our analyses exhibited more recovered events via a pDDT/pgRNA ratio of 4:1 between groups 8 and 9. This ratio caused to increase pDDT dramatically for efficient HDR. The homologous arm lengths were evaluated as the third variable in this study. It has been indicated that the lengths of the homologous arms are vital to making efficient HDR. Our experiments evaluated the homologous arm lengths that exhibited optimized 700 bp compared with the other lengths (**Figure 1E**) (Song and Stieger, 2017). Group 9, with a pDDT/pgRNA ratio of 4:1, Agrobacterium inoculator with OD_600_ = 2.5, and optimized 700 bp homologous arm length, revealed significantly more recovered events than the other groups (**Figure 1E**; **Supplementary Figure 5**). Regarding these findings, Eds_35 transformed with OD_600_ = 2.5, pDDT/pgRNA ratio of 4:1, and 700 bp homologous arm length exhibited a significantly higher expression integrating *nptII* and *MKK2* fusion mRNA expression (**Figure 2B**). Moreover, Eds_35 exhibited the expressions of *MKK2* and its downstream *MKK5* affected by the integrated 2XCamV 35S (**Figure 2D**). Predictably, the fused *MKK2* and exogenous *nptII* mRNA exhibited a relative expression, resulting in an efficient HDR (**Figure 2E**). TaqMan real-time PCR proved that the most HDR efficiency occurred in Ed_35_1, Ed_35_2, and Ed_35_3 (**Figure 3D**). Efficient HDR happened through Ed_35 events causing lower or non-mutant nucleotides leading to significantly decreased polymorphisms in HDR efficiency. The other recovered events from groups 6 and 8 exhibited more polymorphisms (**Figure 3E**).

On the other hand, there were minimum deletion and no insertion mutations through Eds_35 (**Figure 3F**). Further analysis of biochemical and phenotypic changes revealed significantly more total chlorophyll and carotenoids in the Ed_35 recovered events than the other recovered events under 0 and 25 mM of NaCl treatments (**Figure 3G**). The investigation of root length also revealed significantly more growth within Eds_35 than the other recovered edited events under salt stress. Altogether, efficient HDR happened through an efficient transformation with a high concentration of *Agrobacterium* inoculator OD_600_ = 2.5, an increased number of DDTs with a pDDT/pgRNA ratio of 4:1, and a further optimized 700 bp homologous arm length. We concluded that efficient transformation significantly directs efficient HDR, decreasing polymorphisms.

## Conclusion

We have shown that a high concentration of *Agrobacterium* inoculator, homologous arm length optimization, and increasing the number of DDTs via cell division lead to efficient HDR through poplar genome editing. We also proved that a significant reduction in CRISPR-induced polymorphisms could be achieved by following these guidelines. This breakthrough technology will probably encourage biotechnological research, breeding programs, and conservation of tree species and developed crops.

## Data availability statement

All data supporting the findings of this study are available in the article and its Supplementary Figures and Tables. Raw Sanger sequencing data are available on the Mendeley dataset with DOI: 10.17632/5tp44bnczy.3.

## Author contributions

AM: conceptualization, software, formal analysis, writing—original draft, visualization, and project administration. HW: methodology, formal analysis, writing—review and editing, and data curation. SK: validation and writing—review and editing. WS, QZ, and LY: formal analysis and visualization. CX: formal analysis, visualization, and funding acquisition. All authors contributed to the article and approved the submitted version.

## Supplementary material

Supplementary Figure 1 Alignment of Mitogen-activated protein kinase kinase 2 from 14 species to reveal similarities and identify desired sequences in *P. trichocarpa* using Geneious Prime 2022. *Hevea brasiliensis* - XM_021794488.1, *Manihot esculenta* - XR_002487451.1, *Jatropha curcas* - XM_020680666.1, *Populus euphratica* - XM_011018250.1, *Populus trichocarpa* - XM_002324230.2, *Nicotiana tomentosiformis* - XM_009605449.2, *Durio zibethinus* - XM_022899101.1, *Gossypium arboreum* - XM_017790462.1, *Gossypium hirsutum* - NM_001327410.1, *Ziziphus jujuba* - XM_016033841.1, *Juglans regia* - XM_018990646.1, *Betula platyphylla* - KJ459948.1, *Eucalyptus grandis* - XM_010049224.2, *Citrus sinensis* - XM_006475450.2.

Supplementary Figure 2 Phylogenetic tree of Mitogen-activated protein kinase kinase 2; Bootstrap analysis was performed using 1,000 replicates to evaluate the reliability of the various phylogenetic groups.

Supplementary Figure 3 The schematic figures of cassette backbone preparation

Supplementary Figure 4 The schematic figures of homologous arms and pDDT preparation for 500 bp homology sequences

Supplementary Figure 5 Mean comparison of regenerated and recovered events through different designed groups.

Supplementary Figure 6 The raw document used in this research. **(A)** 1457 bp of recombinant genomic DNA has been amplified to prove the proper HDR happenings through recovered events isolated from nptII and *MKK2* locus intron 2. **(B)** 1984 bp of amplified fragments selected from 2XCaMV35S and *MKK2* locus intron 1. On-target and off-target activities are shown as the specific and unspecific bonds, respectively; CT was used as the control. NCT was used as the negative control. Yellow dashed lines indicate cut gels presented in .

Supplementary Figure 7 Sanger sequences, alignment, and sequence analysis from recovered edited events reveal polymorphisms.
