# Supplementary figures and images for: CRISPR-mediated genome editing in poplar issued by efficient transformation
Source: Front Plant Sci. 2023 Apr 17;14:1159615. doi: 10.3389/fpls.2023.1159615 (PMC10149819; doi:10.3389/fpls.2023.1159615)

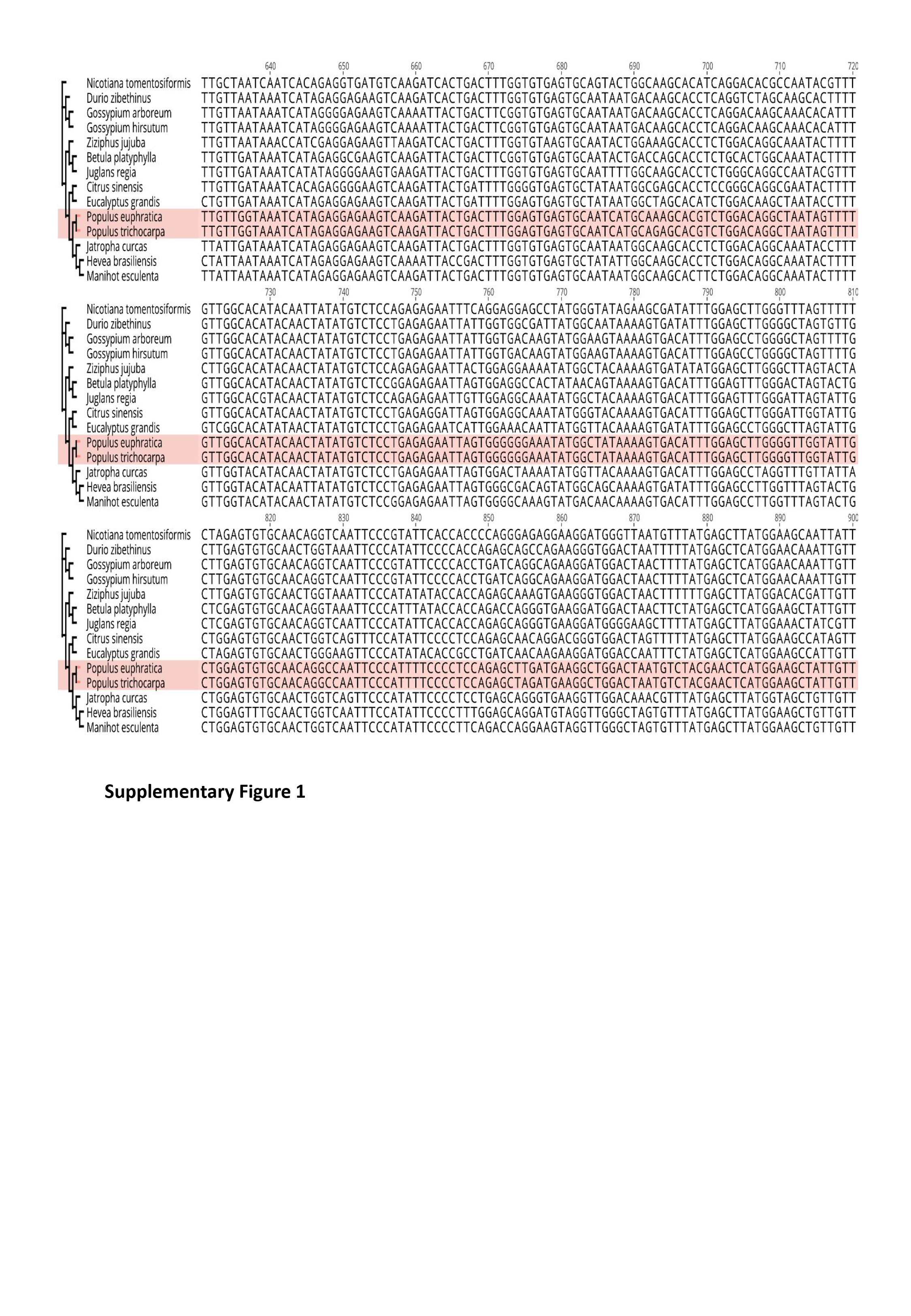

Supplement: Supplementary Figure 1 — Alignment of Mitogen-activated protein kinase kinase 2 from 14 species to reveal similarities and identify desired sequences in P. trichocarpa using Geneious Prime 2022. Hevea brasiliensis - XM_021794488.1, Manihot esculenta - XR_002487451.1, Jatropha curcas - XM_020680666.1, Populus euphratica - XM_011018250.1, Populus trichocarpa - XM_002324230.2, Nicotiana tomentosiformis - XM_009605449.2, Durio zibethinus - XM_022899101.1, Gossypium arboreum - XM_017790462.1, Gossypium hirsutum - NM_001327410.1, Ziziphus jujuba - XM_016033841.1, Juglans regia - XM_018990646.1, Betula platyphylla - KJ459948.1, Eucalyptus grandis - XM_010049224.2, Citrus sinensis - XM_006475450.2. [file Image_1.tif]

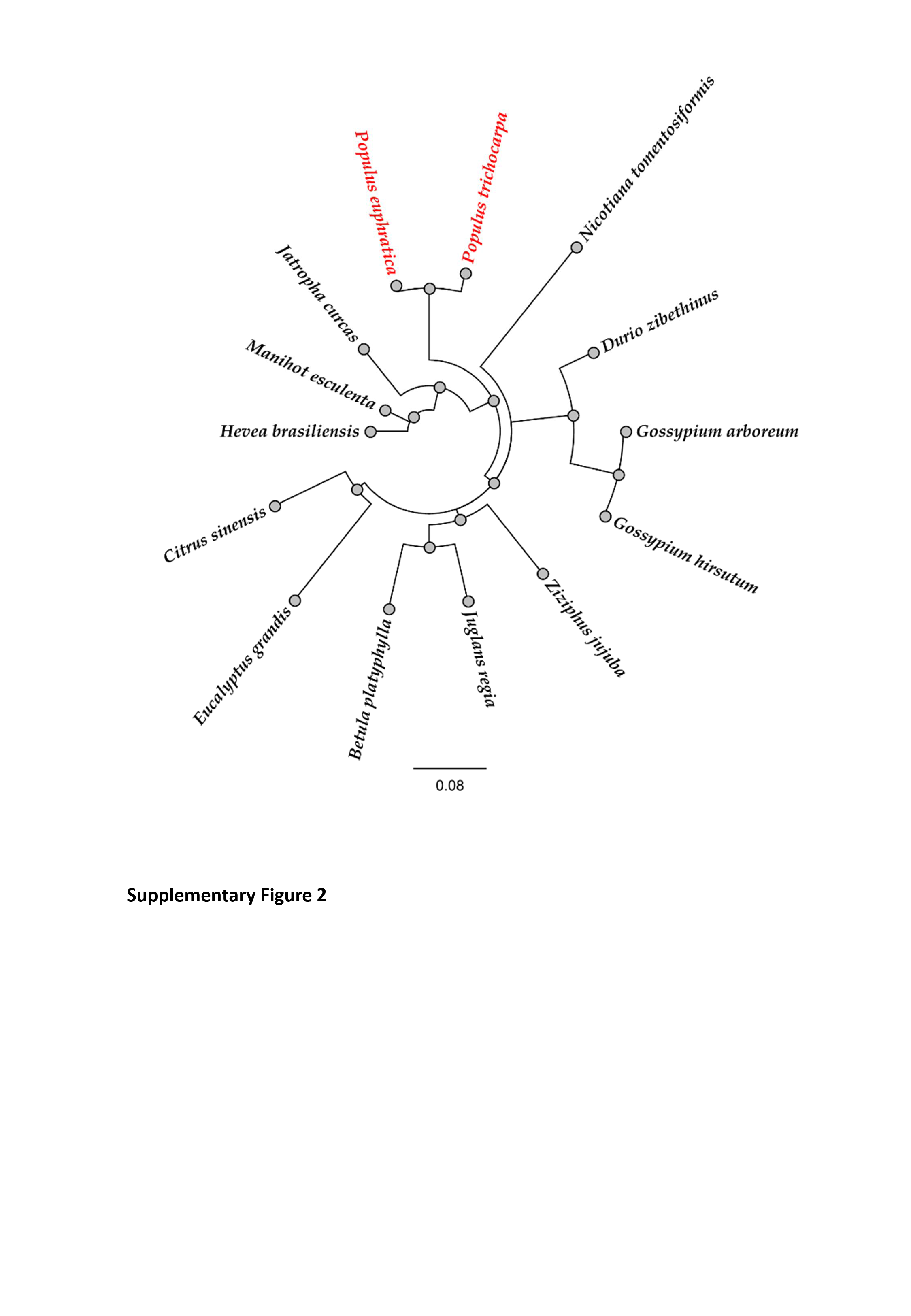

Supplement: Supplementary Figure 2 — Phylogenetic tree of Mitogen-activated protein kinase kinase 2; Bootstrap analysis was performed using 1,000 replicates to evaluate the reliability of the various phylogenetic groups. [file Image_2.tif]

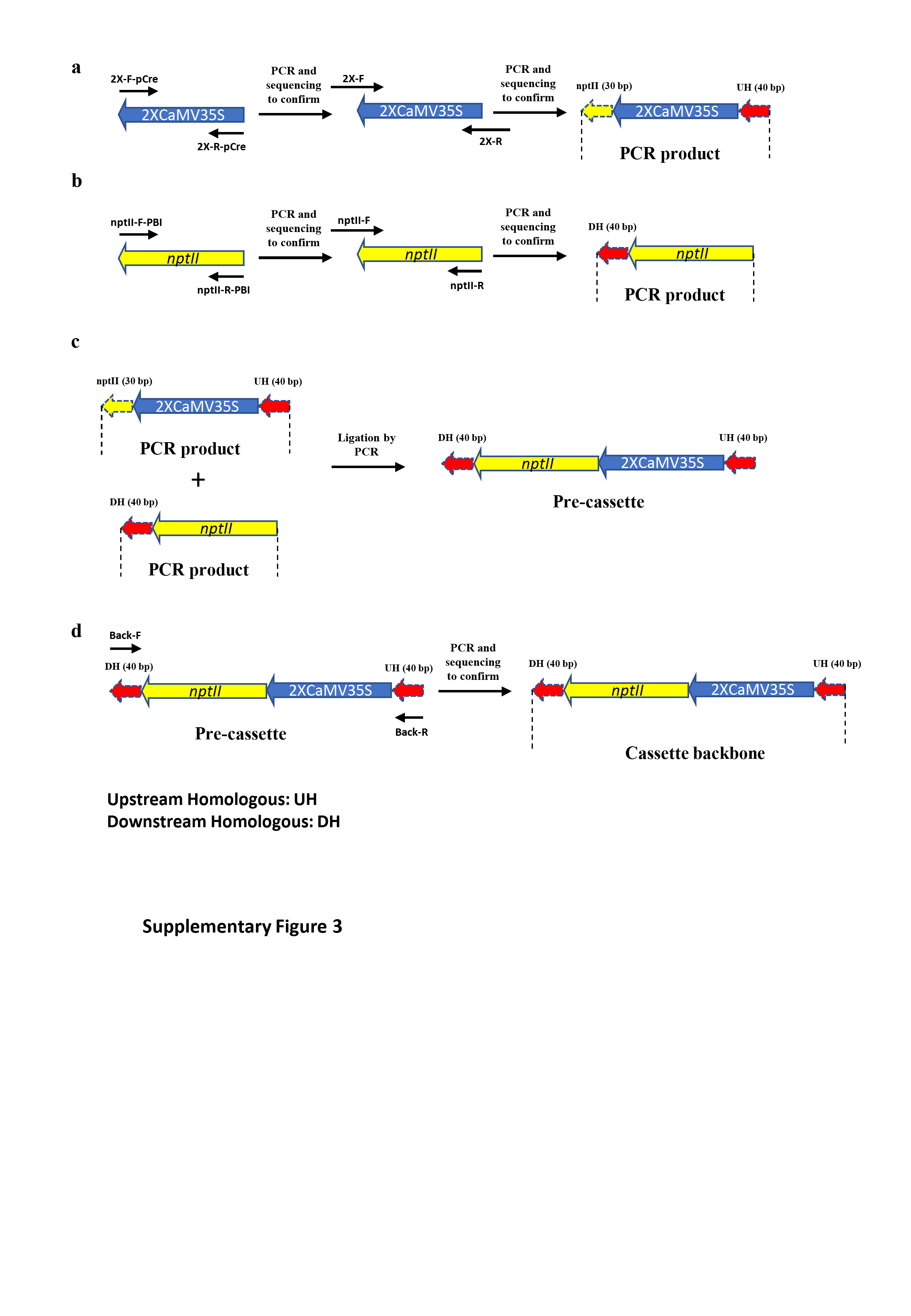

Supplement: Supplementary Figure 3 — The schematic figures of cassette backbone preparation [file Image_3.tif]

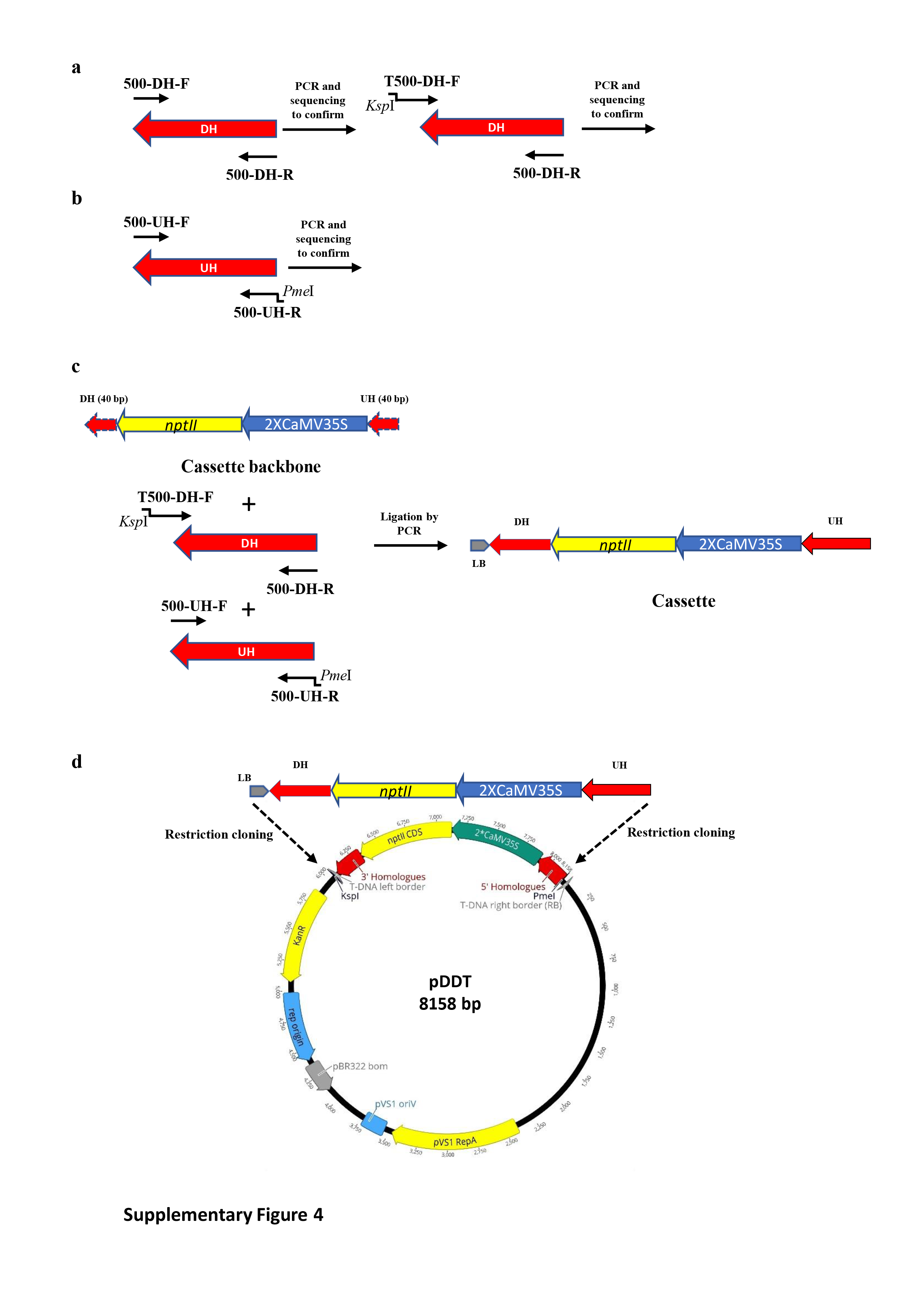

Supplement: Supplementary Figure 4 — The schematic figures of homologous arms and pDDT preparation for 500 bp homology sequences [file Image_4.tif]

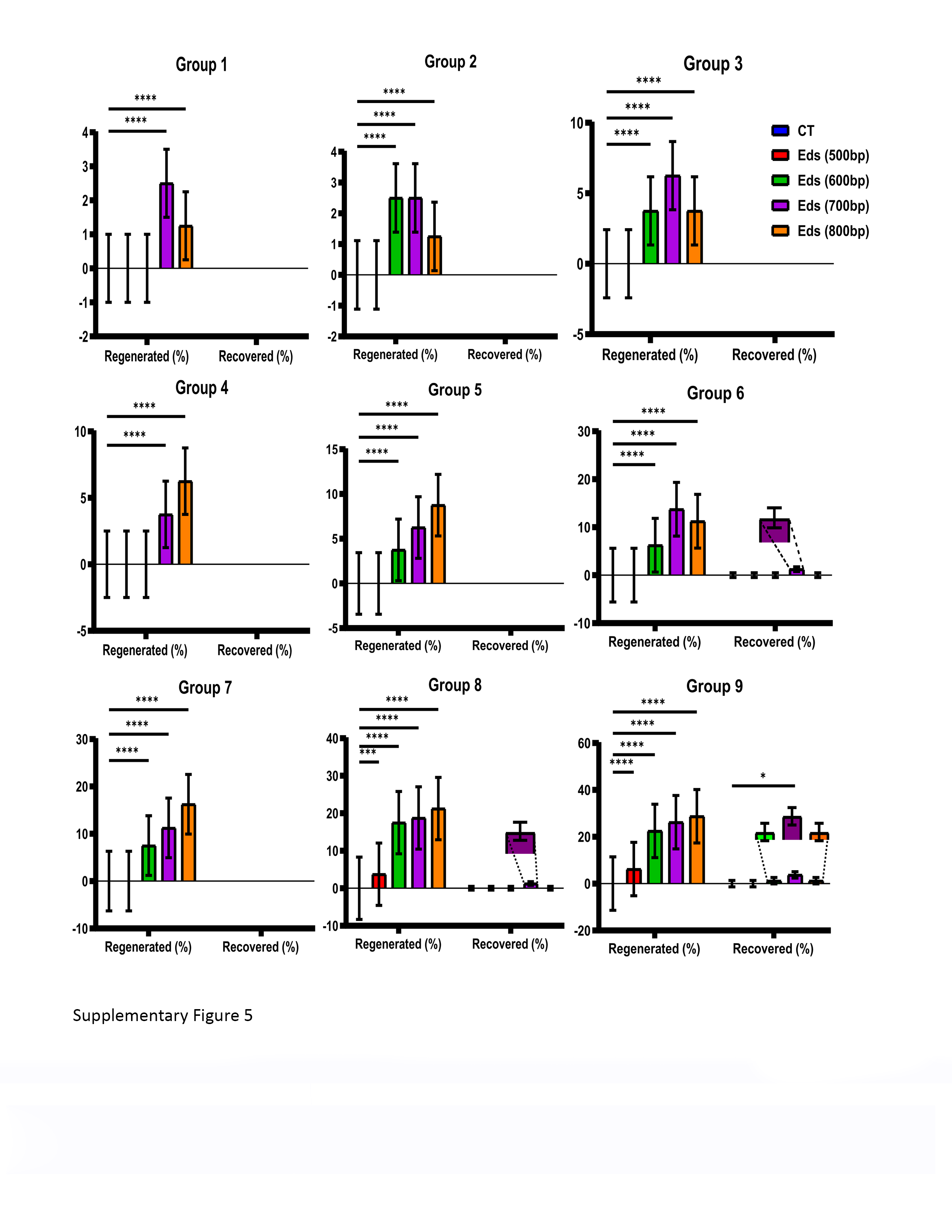

Supplement: Supplementary Figure 5 — Mean comparison of regenerated and recovered events through different designed groups. [file Image_5.tif]

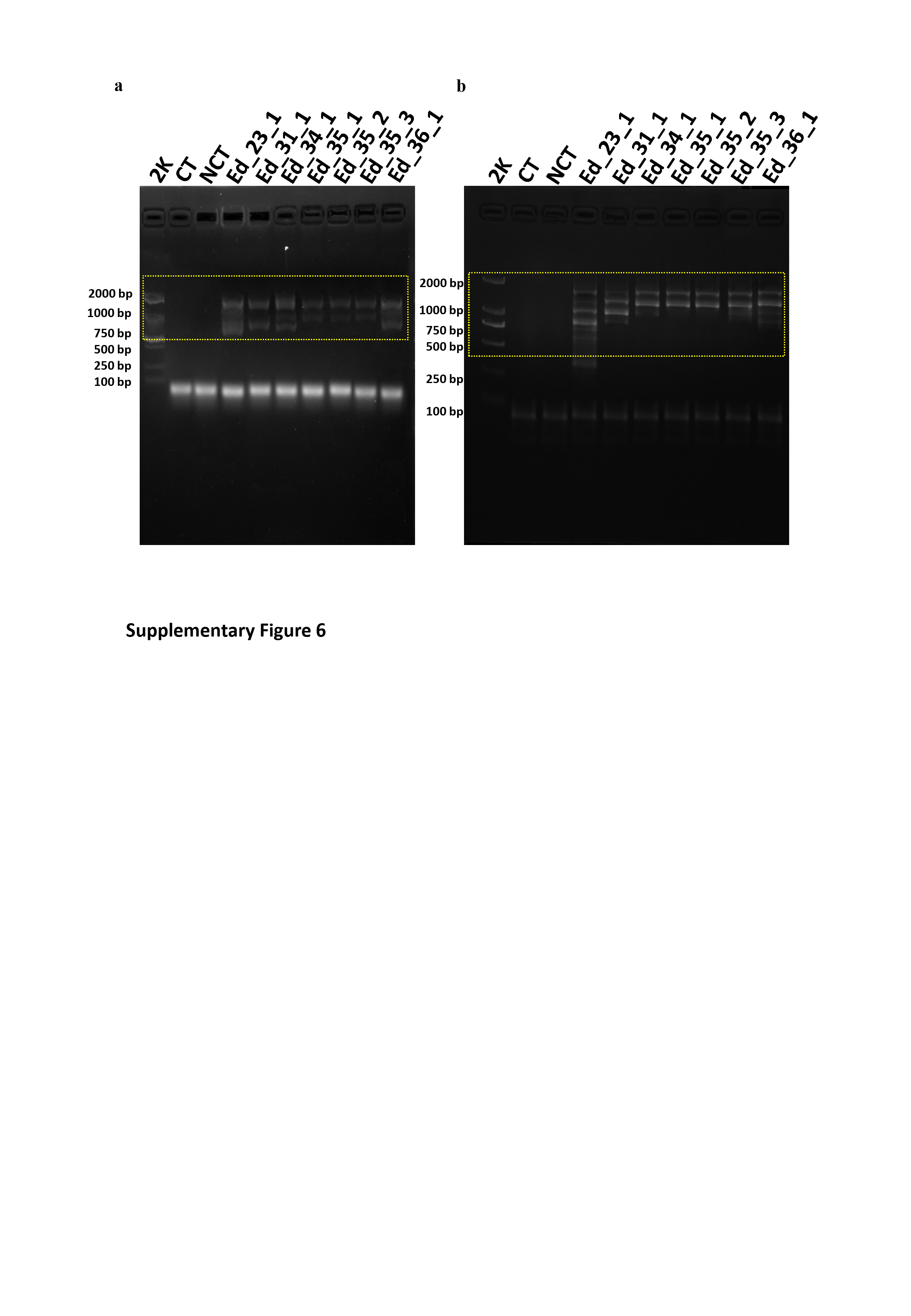

Supplement: Supplementary Figure 6 — The raw document used in this research. (A) 1457 bp of recombinant genomic DNA has been amplified to prove the proper HDR happenings through recovered events isolated from nptII and MKK2 locus intron 2. (B) 1984 bp of amplified fragments selected from 2XCaMV35S and MKK2 locus intron 1. On-target and off-target activities are shown as the specific and unspecific bonds, respectively; CT was used as the control. NCT was used as the negative control. Yellow dashed lines indicate cut gels presented in . [file Image_6.tif]
