# Supplementary material for: CRISPR-mediated genome editing in poplar issued by efficient transformation
Source: Front Plant Sci. 2023 Apr 17;14:1159615. doi: 10.3389/fpls.2023.1159615 (PMC10149819; doi:10.3389/fpls.2023.1159615)
Supplement: Supplementary Figure 7 — Sanger sequences, alignment, and sequence analysis from recovered edited events reveal polymorphisms. [file Image_7.pdf]

```
WT (Reference)            AAAATTACAAGAACTTGGCGAAGGAAAC
                gene
                MAP kinase kinase family protein ...

Ed_23_1                           AAGAACTTGGCGAAGGAAAC
Ed_31_1                           AAGAACTTGGCGAAGGAAAC
Ed_34_1                           AAGAACTTGGCGAAGGAAAC
Ed_35_1                           AAGAACTTGGCGAAGGAAAC
Ed_35_2                           AAGAACTTGGCGAAGGAAAC
Ed_35_3                           AAGAACTTGGCGAAGGAAAC
Ed_36_1         AAGAGGAGAGAAAATTACAAGAACTTGGCGAAGGAAAC

WT (Reference)  TTCATCGGGAGGAGGAAGAGAAAGCTTGAGCTTAACAT
                gene
                MAP kinase kinase family protein mRNA

Ed_23_1         TTCATCGGGAGGAGGAAGAGAAAGCTTGAGCTTAACAT
Ed_31_1         TTCATCGGGAGGAGGAAGAGAAAGCTTGAGCTTAACAT
Ed_34_1         TTCATCGGGAGGAGGAAGAGAAAGCTTGAGCTTAACAT
Ed_35_1         TTCATCGGGAGGAGGAAGAGAAAGCTTGAGCTTAACAT
Ed_35_2         TTCATCGGGAGGAGGAAGAGAAAGCTTGAGCTTAACAT
Ed_35_3         TTCATCGGGAGGAGGAAGAGAAAGCTTGAGCTTAACAT
Ed_36_1         TTCATCGGGAGGAGGAAGAGAAAGCTTGAGCTTAACAT

WT (Reference)  TAGGGTTTAAGCTTCCCTTCTTCATCTTTTGATGAGAT
                gene
                MAP kinase kinase family protein mRNA

Ed_23_1         TAGGGTTTAAGCTTCCCTTCTTCATCTTTTGATGAGAT
Ed_31_1         TAGGGTTTAAGCTTCCCTTCTTCATCTTTTGATGAGAT
Ed_34_1         TAGGGTTTAAGCTTCCCTTCTTCATCTTTTGATGAGAT
Ed_35_1         TAGGGTTTAAGCTTCCCTTCTTCATCTTTTGATGAGAT
Ed_35_2         TAGGGTTTAAGCTTCCCTTCTTCATCTTTTGATGAGAT
Ed_35_3         TAGGGTTTAAGCTTCCCTTCTTCATCTTTTGATGAGAT
Ed_36_1         TAGGGTTTAAGCTTCCCTTCTTCATCTTTTGATGAGAT

WT (Reference)  CTCTTCTCTTCTCTTCTCTTCTCTTTAGGTACGAACAG
                gene
                MAP kinase kinase family protein mRNA

Ed_23_1         CTCTTCTCTTCTCTTCTCTTCTCTTTAGGTACGAACAG
Ed_31_1         CTCTTCTCTTCTCTTCTCTTCTCTTTAGGTACGAACAG
Ed_34_1         CTCTTCTCTTCTCTTCTCTTCTCTTTAGGTACGAACAG
Ed_35_1         CTCTTCTCTTCTCTTCTCTTCTCTTTAGGTACGAACAG
Ed_35_2         CTCTTCTCTTCTCTTCTCTTCTCTTTAGGTACGAACAG
Ed_35_3         CTCTTCTCTTCTCTTCTCTTCTCTTTAGGTACGAACAG
Ed_36_1         CTCTTCTCTTCTCTTCTCTTCTCTTTAGGTACGAACAG

WT (Reference)  CAAAGTCGCTGTAAGTCTTTAAAGAAAAAAAAAATGGT
                gene
                MAP kinase kinase family protein mRNA

Ed_23_1         CAAAGTCGCTGTAA------AAAGAAAAAAAAAATCGT
Ed_31_1         CAAA------GTAAGTCTTTAAAGAAAAAAAAAATGGT
Ed_34_1         CAAAGTCGCTGTAAGTCTTTAAAGAAAAAAAAAATGGT
Ed_35_1         CAAAGTCGCTGTAAGTCTTTAAAGAAAAAAAAAATGGT
Ed_35_2         CAAAGTCGCTGTAAGTCTTTAAAGAAAAAAAAAATGGT
Ed_35_3         CAAAGTCGCTGTAAGTCTTTAAAGAAAAAAAAAATGGT
Ed_36_1         CAAAGTCGCTGTAAGTCTTTAAAGAAAAAAAAAAT---
```

```
WT (Reference)  GGGCTGTGGCTATAATAGACAGTTACTGCATAAAAATT
                gene
                MAP kinase kinase family protein mRNA

Ed_23_1         GGGCTGTGGCTATAATAGACAGTTACTGCATAAAAATT
Ed_31_1         GGGCTGTGGCTATAATAGACAGTTACTGCATAAAAATT
Ed_34_1         GGGCTGTGGCTATAATAGACAGTTACTGCATAAAAATT
Ed_35_1         GGGCTGTGGCTATAATAGACAGTTACTGCATAAAAATT
Ed_35_2         GGGCTGTGGCTATAATAGACAGTTACTGCATAAAAATT
Ed_35_3         GGGCTGTGGCTATAATAGACAGTTACTGCATAAAAATT
Ed_36_1         -GGCTGTGGCTATAATAGACAGTTACTGCATAAAAATT

WT (Reference)  GTTGAAGTAGCAGTAAATGAAAAGGACTATTTATTAAT
                gene
                MAP kinase kinase family protein mRNA

Ed_23_1         GTTGAAGTAGCAGTAAATGAAAAGGACTATTTATTAAT
Ed_31_1         GTTGAAGTAGCAGTAA---AAAAGGACTATTTATTAAT
Ed_34_1         GTTGAAGTAGCAGTAAATGAAAAGGACTATTTATTAA-
Ed_35_1         GTTGAAGTAGCAGTAAATGAAAAGGACTATTTATTAAT
Ed_35_2         GTTGAAGTAGCAGTAAATGAAAAGGACTATTTATTAAT
Ed_35_3         GTTGAAGTAGCAGTAAATGAAAAGGACTATTTATTAAT
Ed_36_1         GTTGAAGTAGCAGTAAATGAAAAGGACTATTTATTAAT

WT (Reference)  TTTCCTTTTTTTTTTTTTTTTGAGGGTGGGGGATGGTA
                gene
                MAP kinase kinase family protein mRNA

Ed_23_1         TTTCCTTTTTTTTTTTTTTTTGAGGGTGGGGGATGGTA
Ed_31_1         --TCCTTTTTTTTTTTTTTTTGAGGGTGGGGGATGGTA
Ed_34_1         TTTCCTTTTTTTTTTTTTTTTGACGGTGGGGGATGGTA
Ed_35_1         TTTCCTTTTTTTTTTTTTTTTGAGGGTGGGGGATGGTA
Ed_35_2         TTTCCTTTTTTTTTTTTTTTTGAGGGTGGGGGATGGTA
Ed_35_3         TTTCCTTTTTTTTTTTTTTTTGAGGGTGGGGGATGGTA
Ed_36_1         TTTCCTTTTTTTTTTTTTTTTGAGGGTGGGGGATGGTA

WT (Reference)  CTTGGTAAACAGCAGTACTAGTCTTTTAGTCTAATCAG
                gene
                MAP kinase kinase family protein mRNA

Ed_23_1         CTTGGTAAACAGCAGTACTAGTCTTTTAGTCTAATCAT
Ed_31_1         CTTGGTAAACAGCAGTACTAGTCTTTTAGTCTAATCAG
Ed_34_1         CTTGGTAAACAGCAGTACTAGTCTTTTAGTCTAATCAG
Ed_35_1         CTTGGTAAACAGCAGTACTAGTCTTTTAGTCTAATCAG
Ed_35_2         CTTGGTAAACAGCAGTACTAGTCTTTTAGTCTAATCAG
Ed_35_3         CTTGGTAAACAGCAGTACTAGTCTTTTAGTCTAATCAG
Ed_36_1         CTTGGTAAACAGCAGTACTAGTCTTTTAGTCTAATCAG

WT (Reference)  TCACCATTACAGAA----ACCACGAAGAA--GATGAAC
                gene
                MAP kinase kinase family protein mRNA

Ed_23_1         TCACCATTACAGAAGAGGACCACGAAGAA--GATGAAC
Ed_31_1         TCACCATTACAGAA----ACCACGAAGAA--GATGAAC
Ed_34_1         TCACCATTACAGAA----ACCACGAAGAA--GATGAAC
Ed_35_1         TCACCATTACAGAA----ACCACGAAGAA--GATGAAC
Ed_35_2         TCACCATTACAGAA----ACCACGAAGAA--GATGAAC
Ed_35_3         TCACCATTACAGAA----ACCACGAAGAA--GATGAAC
Ed_36_1         TCACCATTACAGAA----ACCACGAAGAA--GATGAAC
```

```
WT (Reference)  CTTTCTCTTTCAGTGTTTACTTTTCGTTTTGTTATTTT
                gene
                MAP kinase kinase family protein mRNA

Ed_23_1         CTTTCTCTTTCAGTGTTTACTTTTCGTTTTGTTATTTT
Ed_31_1         CTTTCTCTTTCAGTGTTTACTTTTCGTTTTGTTATTTT
Ed_34_1         CTTTCTCTTTCAGTGTTTACTTTTCGTTTTGTTATTTT
Ed_35_1         CTTTCTCTTTCAGTGTTTACTTTTCGTTTTGTTATTTT
Ed_35_2         CTTTCTCTTTCAGTGTTTACTTTTCGTTTTGTTATTTT
Ed_35_3         CTTTCTCTTTCAGTGTTTACTTTTCGTTTTGTTATTTT
Ed_36_1         CTTTCTCTTTCAGTGTTTACTTTTCGTTTTGTTATTTT

WT (Reference)  TTTATTTCCTGTCTACAGACTGTTTGAT-AAAAAATAT

Ed_23_1         TTTATTTCCTGTCTACAGACTGTTTGATAAAAAAATAT
Ed_31_1         TTTATTTCCTGTCTACAGACTGTTTGAT-AAAAAATAT
Ed_34_1         TTTATTTC---TCTACAGACTGTTTGAT-AAAAAATAT
Ed_35_1         TTTATTTCCTGTCTACAGACTGTTTGAT-AAAAAATAT
Ed_35_2         TTTATTTCCTGTCTACAGACTGTTTGAT-AAAAAATAT
Ed_35_3         TTTATTTCCTGTCTACAGACTGTTTGAT-AAAAAATAT
Ed_36_1         TTTATTTCCTGTCTACAGACTGTTTGAT-AAAAAATAT

WT (Reference)  TTTTTTTGGAAATTTATTAAATAAAT--TTAAAATTTT

Ed_23_1         TTTTTTTGGAAATTTATTAAATAAAT--TTAAAATTTT
Ed_31_1         TTTTTTTGGAAATTTATTAAATAAAT--TTAAAATTTT
Ed_34_1         TTTTTTTGGAAATTTATTAAATAAAT--TTAAAATTTT
Ed_35_1         TTTTTTTGGAAATTTATTAAATAAAT--TTAAAATTTT
Ed_35_2         TTTTTTTGGAAATTTATTAAATAAAT--TTAAAATTTT
Ed_35_3         TTTTTTTG-AAATTTATTAAATAAAT--TTAAAATTTT
Ed_36_1         TTTTTTTGCTTTTTTATTAAATAAAT--TTAAAATTTT

WT (Reference)  ATTTTTAATAGTACAATCAATGAATATATATATATATATA

Ed_23_1         ATTTTTAATAGTACAATCAATGAATATATATATATATATA
Ed_31_1         ATTTTTAATAGTACAATCAATGAATATATATATATATATA
Ed_34_1         ATTTTTAATAGTACAATCAATGAATATATATATATATATA
Ed_35_1         ATTTTTAATAGTACAATCAATGAATATATATATATATATA
Ed_35_2         ATTTTTAATAGTACAATCAATGAATATATATATATATATA
Ed_35_3         ATTTTTAATAGTACAATCAATGAATATATATATATATATA
Ed_36_1         ATTTTTAATAGTACAATCAATGAATATATATATATATATA

WT (Reference)  TATTGAAAATATTTTTTTTTAAAAAAATAATTTTTTTAAA

Ed_23_1         TATTGAAAATATTTTTTTTTAAAAAAATAATTTTTTTAAA
Ed_31_1         T--TGAAAATATTTTTTTTTAAAAAAATAATTTTTTTAAA
Ed_34_1         TATTGAAAATATTTTTTTTTAAAAAAATAATTTTTTTAAA
Ed_35_1         TATTGAAAATATTTTTTTTTAAAAAAATAATTTTTTTAAA
Ed_35_2         TATTGAAAATATTTTTTTTTAAAAAAATAATTTTTTTAAA
Ed_35_3         TATTGAAAATATTTTTTTTTAAAAAAATAATTTTTTTAAA
Ed_36_1         TATTGAAAATATTTTTTTTTAAAAAAATAATTTTTTTAAA
```

```
WT (Reference)    AAAA-CTTTTAAAATAAAAAAACAAATATACCGTTAAA

Ed_23_1           AAAA-CTTTTAAAATAAAAAAACAAATATACCGTTAAA
Ed_31_1           AAAATCTTTTAAAATAAAAAAACAAATATACCGTTAAA
Ed_34_1           AAAA-CTTTTAAAATAAAAAAACAAATATACCGTTAAA
Ed_35_1           AAAA-CTTTTAAAATAAAAAAACAAATATACCGTTAAA
Ed_35_2           AAAA-CTTTTAAAATAAAAAAACAAATATACCGTTAAA
Ed_35_3           AAAA-CTTTTAAAATAAAAAAACAAATATACCGTTAAA
Ed_36_1           AAAA--CTTTAAAATAAAAAAACAAATATACCGTTAAA

WT (Reference)    ACTTATTTATTATAGTAATAATTATTTTTCAAA-TTAA

Ed_23_1           ACTTATTTATTATAGTAATAATTATTTTTCAAA-TTAA
Ed_31_1           ACTTATTTATTATAGTAATAATTATTTTTCAAA-TTAA
Ed_34_1           ACTTATTTATTATAGTAATAATTATTTTTCAAA-TTAA
Ed_35_1           AGATATTTATTATAGTAATAATTATTTTTCAAA-TTAA
Ed_35_2           ACTTATTTATTATAGTAATAATTATTTTTCAAA-TTAA
Ed_35_3           ACTTATTTATTATAGTAATAATTATTTTTCAAA-TTAA
Ed_36_1           ACTTATTTATTATAGTAATAA--ATTTTTCAAA-TTAA

WT (Reference)    TTTTTATTTAAAAACATATCAAAATAATATTTTTTTATT

Ed_23_1           TTTTTATTTAAAAACATATCAAAATAATATTTTTTTATT
Ed_31_1           TTTTTATTTAAAAACATATCAAAATAATATTTTTTTATT
Ed_34_1           TTTTTATTTAAAAACATATCAAAATAATATTTTTTTATT
Ed_35_1           TTTTTATTTAAAAACATATCAAAATAATATTTTTTTATT
Ed_35_2           TTTTTATTTAAAAACATATCAAAATAATATTTTTTTATT
Ed_35_3           TTTTTATTTAAAAACATATCAAAATAATATTTTTTTATT
Ed_36_1           TTTTTATTTAAAAACATATCAAAATAATATTTTTTTATT

WT (Reference)    TTTTAAAAATTTATTTTTAATATAAATAAATTAAAATA

Ed_23_1           TTTTAAAAATTTATTTTTAATATAAATAAATTAAAATA
Ed_31_1           TTTTAAAAATTTATTTTTAATATAAATAAATTAAAATA
Ed_34_1           TTTTAAAAATTTATTTTTAATATAAATAAATTAAAATA
Ed_35_1           TTTTAAAAATTTATTTTTAATATAAATAAATTAAAATA
Ed_35_2           TTTTAAAAATTTATTTTTAATATAAATAAATTAAAATA
Ed_35_3           TTT--AAAATTTATTTCCTATATAAATAAATTAAAATA
Ed_36_1           TTTTAAAAATTTATTTTTAATATAAATAAATTAAAATA

WT (Reference)    ATTTTAAAATACT-AAAAAATTAATTTTAAAAAATTAA

Ed_23_1           ATTTTAAAATACT-AAACCGTTAATTTTAAAAAATTAA
Ed_31_1           ATTTTAAAATACT-AAAAAATTAATTTTAAAAAATTAA
Ed_34_1           ATTTTAAAATACT-AAAAAATTAATTTTAAAAAATTAA
Ed_35_1           ATTTTAAAA--CT-AAAAAATTAATTTTAAAAAATTAA
Ed_35_2           ATTTTAAAA-ACT-AAAAAATTAATTTTAAAAAATTAA
Ed_35_3           ATTTTAAAATACT-AAAAAATTAATTTTAAAAAATTAA
Ed_36_1           ATTTTAAAATACT-AAAAAATTAATTTTAAAAAATTAA
```

```
WT (Reference)  GATTTTTTTTTTTAAAAGAAACAGGCTATTAATATATA

Ed_23_1         GATTTTTTTTTTTAAAAGAAACAGGCTATTAATATATA
Ed_31_1         GATTTTTTTTTTTAAAAGAAACAGGCTATTAATATATA
Ed_34_1         GATTTTTTTTTTTAAAAGAAACAGGCTATTAATATATA
Ed_35_1         GATTTTTTTTTTTAAAAGAAACAGGCTATTAATATATC
Ed_35_2         GATTTTTTTTTTTAAAAGAAACAGGCTATTAATATATA
Ed_35_3         GATTTTTTTTTTTAAAAGAAACAGGCTATTAATATATA
Ed_36_1         GATTTTTTTTTTTAAAAGAAACAGGCTATTAATATATA

WT (Reference)  ATGGGTGGTGATTTCAAGTATTTAAACTAGCAGGTACT

Ed_23_1         ATGGGTGGTGATTTCAAGTATTTAAACTAGCAGGTACT
Ed_31_1         ATGGGTGGTGATTTCAAGTATTTAAACTAGCAGGTACT
Ed_34_1         ATGGGTGGTGGTTTCAAGTATTTAAACTAGCAGGTACT
Ed_35_1         ATGGGTGGTGATTTCAAGTATTTAAACTAGCAGGTACT
Ed_35_2         ATGGGTGGTGATTTCTAGTATTTAAACTAGCAGGTACT
Ed_35_3         ATGGGTGGTGATTTCAAGTATTTAAACTAGCAGGTACT
Ed_36_1         AT-GGTGGTGATTTCAAGTATTTAAACTAGCAGGTACT

WT (Reference)  TCTTCTAACATGTTCAAGTCTTAAGCACGTTACTC---

Ed_23_1         TCTTCTAACATCTTCAGGTCTT-AGCACGTTACTCTCA
Ed_31_1         TCTTCTAACATGTT-AAGTCTTAAGCACGCTACTCTCA
Ed_34_1         TCTTCTAACATGTTCTAGTCTTAAGCACGTTACTCTCA
Ed_35_1         TCTTCTAACATGTTCAAGTCTT-AGCACGTTACTCTCA
Ed_35_2         TCTTCTAACATGTTCAAGTCTTAAGCACGTTACTCTCA
Ed_35_3         TCTTCTAACATGT-CAAGTCTTAAGCACGTTACTCTCA
Ed_36_1         TCTTCTAACATGTTCAAGTCTTAAGGACGT---TCTCA

WT (Reference)  --------------------------------------

Ed_23_1         GAAGAACTCGTCAAGAAGGCGATAGAAGGCGATGCGCT
Ed_31_1         GAAGAACTCGTCAAGAAGGCGATAGAAGGCGATGCGCT
Ed_34_1         GAAGAACTCGTCAAGAAGGCGATAGAAGGCGATGCGCT
Ed_35_1         GAAGAACTCGTCAAGAAGGCGATAGAAGGCGATGCGCT
Ed_35_2         GAAGAACTCGTCAAGAAGGCGATAGAAGGCGATGCGCT
Ed_35_3         GAAGAACTCGTCAAGAAGGCGATAGAAGGCGATGCGCT
Ed_36_1         GAAGAACTCGTCAAGAAGGCGATAGAAGGCGATGCGCT

WT (Reference)  --------------------------------------

Ed_23_1         GCGAACGGGAGCGGCGATACCGTAAAGCACGAGGAAGC
Ed_31_1         GCGAACGGGAGCGGCGATACCGTAAAGCACGAGGAAGC
Ed_34_1         GCGAACGGGAGCGGCGATACCGTAAAGCACGAGGAAGC
Ed_35_1         GCGAACGGGAGCGGCGATACCGTAAAGCACGAGGAAGC
Ed_35_2         GCGAACGGGAGCGGCGATACCGTAAAGCACGAGGAAGC
Ed_35_3         GCGAACGGGAGCGGCGATACCGTAAAGCACGAGGAAGC
Ed_36_1         GCGAACGGGAGCGGCGATACCGTAAAGCACGAGGAAGC
```

```
WT (Reference)  --------------------------------------

Ed_23_1         GGTCAGCCCATTCGCCGCCAAGCTCTTCAGCAATATCA
Ed_31_1         GGTCAGCCCATTCGCCGCCAAGCTCTTCAGCAATATCA
Ed_34_1         GGTCAGCCCATTCGCCGCCAAGCTCTTCAGCAATATCA
Ed_35_1         GGTCAGCCCATTCGCCGCCAAGCTCTTCAGCAATATCA
Ed_35_2         GGTCAGCCCATTCGCCGCCAAGCTCTTCAGCAATATCA
Ed_35_3         GGTCAGCCCATTCGCCGCCAAGCTCTTCAGCAATATCA
Ed_36_1         GGTCAGCCCATTCGCCGCCAAGCTCTTCAGCAATATCA

WT (Reference)  --------------------------------------

Ed_23_1         CGGGTAGCCAACGCTATGTCCTGATAGCGGTCCGCCAC
Ed_31_1         CGGGTAGCCAACGCTATGTCCTGATAGCGGTCCGCCAC
Ed_34_1         CGGGTAGCCAACGCTATGTCCTGATAGCGGTCCGCCAC
Ed_35_1         CGGGTAGCCAACGCTATGTCCTGATAGCGGTCCGCCAC
Ed_35_2         CGGGTAGCCAACGCTATGTCCTGATAGCGGTCCGCCAC
Ed_35_3         CGGGTAGCCAACGCTATGTCCTGATAGCGGTCCGCCAC
Ed_36_1         CGGGTAGCCAACGCTATGTCCTGATAGCGGTCCGCCAC

WT (Reference)  --------------------------------------

Ed_23_1         ACCCAGCCGGCCACAGTCGATGAATCCAGAAAAGCGGC
Ed_31_1         ACCCAGCCGGCCACAGTCGATGAATCCAGAAAAGCGGC
Ed_34_1         ACCCAGCCGGCCACAGTCGATGAATCCAGAAAAGCGGC
Ed_35_1         ACCCAGCCGGCCACAGTCGATGAATCCAGAAAAGCGGC
Ed_35_2         ACCCAGCCGGCCACAGTCGATGAATCCAGAAAAGCGGC
Ed_35_3         ACCCAGCCGGCCACAGTCGATGAATCCAGAAAAGCGGC
Ed_36_1         ACCCAGCCGGCCACAGTCGATGAATCCAGAAAAGCGGC

WT (Reference)  --------------------------------------

Ed_23_1         CATTTTCCACCATGATATTCGGCAAGCAGGCATCGCCA
Ed_31_1         CATTTTCCACCATGATATTCGGCAAGCAGGCATCGCCA
Ed_34_1         CATTTTCCACCATGATATTCGGCAAGCAGGCATCGCCA
Ed_35_1         CATTTTCCACCATGATATTCGGCAAGCAGGCATCGCCA
Ed_35_2         CATTTTCCACCATGATATTCGGCAAGCAGGCATCGCCA
Ed_35_3         CATTTTCCACCATGATATTCGGCAAGCAGGCATCGCCA
Ed_36_1         CATTTTCCACCATGATATTCGGCAAGCAGGCATCGCCA

WT (Reference)  --------------------------------------

Ed_23_1         TGGGTCACGACGAGATCATCGCCGTCGGGCATGCGCGC
Ed_31_1         TGGGTCACGACGAGATCATCGCCGTCGGGCATGCGCGC
Ed_34_1         TGGGTCACGACGAGATCATCGCCGTCGGGCATGCGCGC
Ed_35_1         TGGGTCACGACGAGATCATCGCCGTCGGGCATGCGCGC
Ed_35_2         TGGGTCACGACGAGATCATCGCCGTCGGGCATGCGCGC
Ed_35_3         TGGGTCACGACGAGATCATCGCCGTCGGGCATGCGCGC
Ed_36_1         TGGGTCACGACGAGATCATCGCCGTCGGGCATGCGCGC
```

```
WT (Reference)  --------------------------------------

Ed_23_1         CTTGAGCCTGGCGAACAGTTCGGCTGGCGCGAGCCCCT
Ed_31_1         CTTGAGCCTGGCGAACAGTTCGGCTGGCGCGAGCCCCT
Ed_34_1         CTTGAGCCTGGCGAACAGTTCGGCTGGCGCGAGCCCCT
Ed_35_1         CTTGAGCCTGGCGAACAGTTCGGCTGGCGCGAGCCCCT
Ed_35_2         CTTGAGCCTGGCGAACAGTTCGGCTGGCGCGAGCCCCT
Ed_35_3         CTTGAGCCTGGCGAACAGTTCGGCTGGCGCGAGCCCCT
Ed_36_1         CTTGAGCCTGGCGAACAGTTCGGCTGGCGCGAGCCCCT

WT (Reference)  --------------------------------------

Ed_23_1         GATGCTCTTCGTCCAGATCATCCTGATCGACAAGACCG
Ed_31_1         GATGCTCTTCGTCCAGATCATCCTGATCGACAAGACCG
Ed_34_1         GATGCTCTTCGTCCAGATCATCCTGATCGACAAGACCG
Ed_35_1         GATGCTCTTCGTCCAGATCATCCTGATCGACAAGACCG
Ed_35_2         GATGCTCTTCGTCCAGATCATCCTGATCGACAAGACCG
Ed_35_3         GATGCTCTTCGTCCAGATCATCCTGATCGACAAGACCG
Ed_36_1         GATGCTCTTCGTCCAGATCATCCTGATCGACAAGACCG

WT (Reference)  --------------------------------------

Ed_23_1         GCTTCCATCCGAGTACGTGCTCGCTCGATGCGATGTTT
Ed_31_1         GCTTCCATCCGAGTACGTGCTCGCTCGATGCGATGTTT
Ed_34_1         GCTTCCATCCGAGTACGTGCTCGCTCGATGCGATGTTT
Ed_35_1         GCTTCCATCCGAGTACGTGCTCGCTCGATGCGATGTTT
Ed_35_2         GCTTCCATCCGAGTACGTGCTCGCTCGATGCGATGTTT
Ed_35_3         GCTTCCATCCGAGTACGTGCTCGCTCGATGCGATGTTT
Ed_36_1         GCTTCCATCCGAGTACGTGCTCGCTCGATGCGATGTTT

WT (Reference)  --------------------------------------

Ed_23_1         CGCTTGGTGGTCGAATGGGCAGGTAGCCGGATCAAGCG
Ed_31_1         CGCTTGGTGGTCGAATGGGCAGGTAGCCGGATCAAGCG
Ed_34_1         CGCTTGGTGGTCGAATGGGCAGGTAGCCGGATCAAGCG
Ed_35_1         CGCTTGGTGGTCGAATGGGCAGGTAGCCGGATCAAGCG
Ed_35_2         CGCTTGGTGGTCGAATGGGCAGGTAGCCGGATCAAGCG
Ed_35_3         CGCTTGGTGGTCGAATGGGCAGGTAGCCGGATCAAGCG
Ed_36_1         CGCTTGGTGGTCGAATGGGCAGGTAGCCGGATCAAGCG

WT (Reference)  --------------------------------------

Ed_23_1         TATGCAGCCGCCGCATTGCATCAGCCATGATGGATACT
Ed_31_1         TATGCAGCCGCCGCATTGCATCAGCCATGATGGATACT
Ed_34_1         TATGCAGCCGCCGCATTGCATCAGCCATGATGGATACT
Ed_35_1         TATGCAGCCGCCGCATTGCATCAGCCATGATGGATACT
Ed_35_2         TATGCAGCCGCCGCATTGCATCAGCCATGATGGATACT
Ed_35_3         TATGCAGCCGCCGCATTGCATCAGCCATGATGGATACT
Ed_36_1         TATGCAGCCGCCGCATTGCATCAGCCATGATGGATACT
```

```
WT (Reference)   --------------------------------------

Ed_23_1          TTCTCGGCAGGAGCAAGGTGAGATGACAGGAGATCCTG
Ed_31_1          TTCTCGGCAGGAGCAAGGTGAGATGACAGGAGATCCTG
Ed_34_1          TTCTCGGCAGGAGCAAGGTGAGATGACAGGAGATCCTG
Ed_35_1          TTCTCGGCAGGAGCAAGGTGAGATGACAGGAGATCCTG
Ed_35_2          TTCTCGGCAGGAGCAAGGTGAGATGACAGGAGATCCTG
Ed_35_3          TTCTCGGCAGGAGCAAGGTGAGATGACAGGAGATCCTG
Ed_36_1          TTCTCGGCAGGAGCAAGGTGAGATGACAGGAGATCCTG

WT (Reference)   --------------------------------------

Ed_23_1          CCCCGGCACTTCGCCCAATAGCAGCCAGTCCCTTCCCG
Ed_31_1          CCCCGGCACTTCGCCCAATAGCAGCCAGTCCCTTCCCG
Ed_34_1          CCCCGGCACTTCGCCCAATAGCAGCCAGTCCCTTCCCG
Ed_35_1          CCCCGGCACTTCGCCCAATAGCAGCCAGTCCCTTCCCG
Ed_35_2          CCCCGGCACTTCGCCCAATAGCAGCCAGTCCCTTCCCG
Ed_35_3          CCCCGGCACTTCGCCCAATAGCAGCCAGTCCCTTCCCG
Ed_36_1          CCCCGGCACTTCGCCCAATAGCAGCCAGTCCCTTCCCG

WT (Reference)   --------------------------------------

Ed_23_1          CTTCAGTGACAACGTCGAGCACAGCTGCGCAAGGAACG
Ed_31_1          CTTCAGTGACAACGTCGAGCACAGCTGCGCAAGGAACG
Ed_34_1          CTTCAGTGACAACGTCGAGCACAGCTGCGCAAGGAACG
Ed_35_1          CTTCAGTGACAACGTCGAGCACAGCTGCGCAAGGAACG
Ed_35_2          CTTCAGTGACAACGTCGAGCACAGCTGCGCAAGGAACG
Ed_35_3          CTTCAGTGACAACGTCGAGCACAGCTGCGCAAGGAACG
Ed_36_1          CTTCAGTGACAACGTCGAGCACAGCTGCGCAAGGAACG

WT (Reference)   --------------------------------------

Ed_23_1          CCCGTCGTGGCCAGCCACGATAGCCGCGCTGCCTCGTC
Ed_31_1          CCCGTCGTGGCCAGCCACGATAGCCGCGCTGCCTCGTC
Ed_34_1          CCCGTCGTGGCCAGCCACGATAGCCGCGCTGCCTCGTC
Ed_35_1          CCCGTCGTGGCCAGCCACGATAGCCGCGCTGCCTCGTC
Ed_35_2          CCCGTCGTGGCCAGCCACGATAGCCGCGCTGCCTCGTC
Ed_35_3          CCCGTCGTGGCCAGCCACGATAGCCGCGCTGCCTCGTC
Ed_36_1          CCCGTCGTGGCCAGCCACGATAGCCGCGCTGCCTCGTC

WT (Reference)   --------------------------------------

Ed_23_1          CTGCAGTTCATTCAGGGCACCGGACAGGTCGGTCTTGA
Ed_31_1          CTGCAGTTCATTCAGGGCACCGGACAGGTCGGTCTTGA
Ed_34_1          CTGCAGTTCATTCAGGGCACCGGACAGGTCGGTCTTGA
Ed_35_1          CTGCAGTTCATTCAGGGCACCGGACAGGTCGGTCTTGA
Ed_35_2          CTGCAGTTCATTCAGGGCACCGGACAGGTCGGTCTTGA
Ed_35_3          CTGCAGTTCATTCAGGGCACCGGACAGGTCGGTCTTGA
Ed_36_1          CTGCAGTTCATTCAGGGCACCGGACAGGTCGGTCTTGA
```

```
WT (Reference)  --------------------------------------

Ed_23_1         CAAAAAGAACCGGGCGCCCCTGCGCTGACAGCCGGAAC
Ed_31_1         CAAAAAGAACCGGGCGCCCCTGCGCTGACAGCCGGAAC
Ed_34_1         CAAAAAGAACCGGGCGCCCCTGCGCTGACAGCCGGAAC
Ed_35_1         CAAAAAGAACCGGGCGCCCCTGCGCTGACAGCCGGAAC
Ed_35_2         CAAAAAGAACCGGGCGCCCCTGCGCTGACAGCCGGAAC
Ed_35_3         CAAAAAGAACCGGGCGCCCCTGCGCTGACAGCCGGAAC
Ed_36_1         CAAAAAGAACCGGGCGCCCCTGCGCTGACAGCCGGAAC

WT (Reference)  --------------------------------------

Ed_23_1         ACGGCGGCATCAGAGCAGCCGATTGTCTGTTGTGCCCA
Ed_31_1         ACGGCGGCATCAGAGCAGCCGATTGTCTGTTGTGCCCA
Ed_34_1         ACGGCGGCATCAGAGCAGCCGATTGTCTGTTGTGCCCA
Ed_35_1         ACGGCGGCATCAGAGCAGCCGATTGTCTGTTGTGCCCA
Ed_35_2         ACGGCGGCATCAGAGCAGCCGATTGTCTGTTGTGCCCA
Ed_35_3         ACGGCGGCATCAGAGCAGCCGATTGTCTGTTGTGCCCA
Ed_36_1         ACGGCGGCATCAGAGCAGCCGATTGTCTGTTGTGCCCA

WT (Reference)  --------------------------------------

Ed_23_1         GTCATAGCCGAATAGCCTCTCCACCCAAGCGGCCGGAG
Ed_31_1         GTCATAGCCGAATAGCCTCTCCACCCAAGCGGCCGGAG
Ed_34_1         GTCATAGCCGAATAGCCTCTCCACCCAAGCGGCCGGAG
Ed_35_1         GTCATAGCCGAATAGCCTCTCCACCCAAGCGGCCGGAG
Ed_35_2         GTCATAGCCGAATAGCCTCTCCACCCAAGCGGCCGGAG
Ed_35_3         GTCATAGCCGAATAGCCTCTCCACCCAAGCGGCCGGAG
Ed_36_1         GTCATAGCCGAATAGCCTCTCCACCCAAGCGGCCGGAG

WT (Reference)  --------------------------------------

Ed_23_1         AACCTGCGTGCAATCCATCTTGTTCAATCATAGAGAGA
Ed_31_1         AACCTGCGTGCAATCCATCTTGTTCAATCATAGAGAGA
Ed_34_1         AACCTGCGTGCAATCCATCTTGTTCAATCATAGAGAGA
Ed_35_1         AACCTGCGTGCAATCCATCTTGTTCAATCATAGAGAGA
Ed_35_2         AACCTGCGTGCAATCCATCTTGTTCAATCATAGAGAGA
Ed_35_3         AACCTGCGTGCAATCCATCTTGTTCAATCATAGAGAGA
Ed_36_1         AACCTGCGTGCAATCCATCTTGTTCAATCATAGAGAGA

WT (Reference)  --------------------------------------

Ed_23_1         CTGGTGATTTCAGCGTGTCCTCTCCAAATGAAATGAAC
Ed_31_1         CTGGTGATTTCAGCGTGTCCTCTCCAAATGAAATGAAC
Ed_34_1         CTGGTGATTTCAGCGTGTCCTCTCCAAATGAAATGAAC
Ed_35_1         CTGGTGATTTCAGCGTGTCCTCTCCAAATGAAATGAAC
Ed_35_2         CTGGTGATTTCAGCGTGTCCTCTCCAAATGAAATGAAC
Ed_35_3         CTGGTGATTTCAGCGTGTCCTCTCCAAATGAAATGAAC
Ed_36_1         CTGGTGATTTCAGCGTGTCCTCTCCAAATGAAATGAAC
```

```
WT (Reference)    --------------------------------------

Ed_23_1           TTCCTTATATAGAGGAAGGTCTTGCGAAGGATAGTGGG
Ed_31_1           TTCCTTATATAGAGGAAGGTCTTGCGAAGGATAGTGGG
Ed_34_1           TTCCTTATATAGAGGAAGGTCTTGCGAAGGATAGTGGG
Ed_35_1           TTCCTTATATAGAGGAAGGTCTTGCGAAGGATAGTGGG
Ed_35_2           TTCCTTATATAGAGGAAGGTCTTGCGAAGGATAGTGGG
Ed_35_3           TTCCTTATATAGAGGAAGGTCTTGCGAAGGATAGTGGG
Ed_36_1           TTCCTTATATAGAGGAAGGTCTTGCGAAGGATAGTGGG

WT (Reference)    --------------------------------------

Ed_23_1           ATTGTGCGTCATCCCTTACGTCAGTGGAGATATCACAT
Ed_31_1           ATTGTGCGTCATCCCTTACGTCAGTGGAGATATCACAT
Ed_34_1           ATTGTGCGTCATCCCTTACGTCAGTGGAGATATCACAT
Ed_35_1           ATTGTGCGTCATCCCTTACGTCAGTGGAGATATCACAT
Ed_35_2           ATTGTGCGTCATCCCTTACGTCAGTGGAGATATCACAT
Ed_35_3           ATTGTGCGTCATCCCTTACGTCAGTGGAGATATCACAT
Ed_36_1           ATTGTGCGTCATCCCTTACGTCAGTGGAGATATCACAT

WT (Reference)    --------------------------------------

Ed_23_1           CAATCCACTTGCTTTGAAGACGTGGTTGGAACGTCTTC
Ed_31_1           CAATCCACTTGCTTTGAAGACGTGGTTGGAACGTCTTC
Ed_34_1           CAATCCACTTGCTTTGAAGACGTGGTTGGAACGTCTTC
Ed_35_1           CAATCCACTTGCTTTGAAGACGTGGTTGGAACGTCTTC
Ed_35_2           CAATCCACTTGCTTTGAAGACGTGGTTGGAACGTCTTC
Ed_35_3           CAATCCACTTGCTTTGAAGACGTGGTTGGAACGTCTTC
Ed_36_1           CAATCCACTTGCTTTGAAGACGTGGTTGGAACGTCTTC

WT (Reference)    --------------------------------------

Ed_23_1           TTTTTCCACGATGCTCCTCGTGGGTGGGGGTCCATCTT
Ed_31_1           TTTTTCCACGATGCTCCTCGTGGGTGGGGGTCCATCTT
Ed_34_1           TTTTTCCACGATGCTCCTCGTGGGTGGGGGTCCATCTT
Ed_35_1           TTTTTCCACGATGCTCCTCGTGGGTGGGGGTCCATCTT
Ed_35_2           TTTTTCCACGATGCTCCTCGTGGGTGGGGGTCCATCTT
Ed_35_3           TTTTTCCACGATGCTCCTCGTGGGTGGGGGTCCATCTT
Ed_36_1           TTTTTCCACGATGCTCCTCGTGGGTGGGGGTCCATCTT

WT (Reference)    --------------------------------------

Ed_23_1           TGGGACCACTGTCGGCAGAGGCATCTTGAACGATAGCC
Ed_31_1           TGGGACCACTGTCGGCAGAGGCATCTTGAACGATAGCC
Ed_34_1           TGGGACCACTGTCGGCAGAGGCATCTTGAACGATAGCC
Ed_35_1           TGGGACCACTGTCGGCAGAGGCATCTTGAACGATAGCC
Ed_35_2           TGGGACCACTGTCGGCAGAGGCATCTTGAACGATAGCC
Ed_35_3           TGGGACCACTGTCGGCAGAGGCATCTTGAACGATAGCC
Ed_36_1           TGGGACCACTGTCGGCAGAGGCATCTTGAACGATAGCC
```

```
WT (Reference)  --------------------------------------

Ed_23_1         TTTCCTTTATCGCAATGATGGCATTTGTAGGTGCCACC
Ed_31_1         TTTCCTTTATCGCAATGATGGCATTTGTAGGTGCCACC
Ed_34_1         TTTCCTTTATCGCAATGATGGCATTTGTAGGTGCCACC
Ed_35_1         TTTCCTTTATCGCAATGATGGCATTTGTAGGTGCCACC
Ed_35_2         TTTCCTTTATCGCAATGATGGCATTTGTAGGTGCCACC
Ed_35_3         TTTCCTTTATCGCAATGATGGCATTTGTAGGTGCCACC
Ed_36_1         TTTCCTTTATCGCAATGATGGCATTTGTAGGTGCCACC

WT (Reference)  --------------------------------------

Ed_23_1         TTCCTTTTCTACTGTCCTTTTGATGAAGTGACAGATAG
Ed_31_1         TTCCTTTTCTACTGTCCTTTTGATGAAGTGACAGATAG
Ed_34_1         TTCCTTTTCTACTGTCCTTTTGATGAAGTGACAGATAG
Ed_35_1         TTCCTTTTCTACTGTCCTTTTGATGAAGTGACAGATAG
Ed_35_2         TTCCTTTTCTACTGTCCTTTTGATGAAGTGACAGATAG
Ed_35_3         TTCCTTTTCTACTGTCCTTTTGATGAAGTGACAGATAG
Ed_36_1         TTCCTTTTCTACTGTCCTTTTGATGAAGTGACAGATAG

WT (Reference)  --------------------------------------

Ed_23_1         CTGGGCAATGGAATCCGAGGAGGTTTCCCGATATTACC
Ed_31_1         CTGGGCAATGGAATCCGAGGAGGTTTCCCGATATTACC
Ed_34_1         CTGGGCAATGGAATCCGAGGAGGTTTCCCGATATTACC
Ed_35_1         CTGGGCAATGGAATCCGAGGAGGTTTCCCGATATTACC
Ed_35_2         CTGGGCAATGGAATCCGAGGAGGTTTCCCGATATTACC
Ed_35_3         CTGGGCAATGGAATCCGAGGAGGTTTCCCGATATTACC
Ed_36_1         CTGGGCAATGGAATCCGAGGAGGTTTCCCGATATTACC

WT (Reference)  --------------------------------------

Ed_23_1         CTTTGTTGAAAAGTCTCAATAGCCCTTTGGTCTTCTGA
Ed_31_1         CTTTGTTGAAAAGTCTCAATAGCCCTTTGGTCTTCTGA
Ed_34_1         CTTTGTTGAAAAGTCTCAATAGCCCTTTGGTCTTCTGA
Ed_35_1         CTTTGTTGAAAAGTCTCAATAGCCCTTTGGTCTTCTGA
Ed_35_2         CTTTGTTGAAAAGTCTCAATAGCCCTTTGGTCTTCTGA
Ed_35_3         CTTTGTTGAAAAGTCTCAATAGCCCTTTGGTCTTCTGA
Ed_36_1         CTTTGTTGAAAAGTCTCAATAGCCCTTTGGTCTTCTGA

WT (Reference)  --------------------------------------

Ed_23_1         GACTGTATCTTTGATATTCTTGGAGTAGACGAGAGTGT
Ed_31_1         GACTGTATCTTTGATATTCTTGGAGTAGACGAGAGTGT
Ed_34_1         GACTGTATCTTTGATATTCTTGGAGTAGACGAGAGTGT
Ed_35_1         GACTGTATCTTTGATATTCTTGGAGTAGACGAGAGTGT
Ed_35_2         GACTGTATCTTTGATATTCTTGGAGTAGACGAGAGTGT
Ed_35_3         GACTGTATCTTTGATATTCTTGGAGTAGACGAGAGTGT
Ed_36_1         GACTGTATCTTTGATATTCTTGGAGTAGACGAGAGTGT
```

```
WT (Reference)  --------------------------------------

Ed_23_1         CGTGCTCCACCATGTTATCACATCAATCCACTTGCTTT
Ed_31_1         CGTGCTCCACCATGTTATCACATCAATCCACTTGCTTT
Ed_34_1         CGTGCTCCACCATGTTATCACATCAATCCACTTGCTTT
Ed_35_1         CGTGCTCCACCATGTTATCACATCAATCCACTTGCTTT
Ed_35_2         CGTGCTCCACCATGTTATCACATCAATCCACTTGCTTT
Ed_35_3         CGTGCTCCACCATGTTATCACATCAATCCACTTGCTTT
Ed_36_1         CGTGCTCCACCATGTTATCACATCAATCCACTTGCTTT

WT (Reference)  --------------------------------------

Ed_23_1         GAAGACGTGGTTGGAACGTCTTCTTTTTCCACGATGCT
Ed_31_1         GAAGACGTGGTTGGAACGTCTTCTTTTTCCACGATGCT
Ed_34_1         GAAGACGTGGTTGGAACGTCTTCTTTTTCCACGATGCT
Ed_35_1         GAAGACGTGGTTGGAACGTCTTCTTTTTCCACGATGCT
Ed_35_2         GAAGACGTGGTTGGAACGTCTTCTTTTTCCACGATGCT
Ed_35_3         GAAGACGTGGTTGGAACGTCTTCTTTTTCCACGATGCT
Ed_36_1         GAAGACGTGGTTGGAACGTCTTCTTTTTCCACGATGCT

WT (Reference)  --------------------------------------

Ed_23_1         CCTCGTGGGTGGGGGTCCATCTTTGGGACCACTGTCGG
Ed_31_1         CCTCGTGGGTGGGGGTCCATCTTTGGGACCACTGTCGG
Ed_34_1         CCTCGTGGGTGGGGGTCCATCTTTGGGACCACTGTCGG
Ed_35_1         CCTCGTGGGTGGGGGTCCATCTTTGGGACCACTGTCGG
Ed_35_2         CCTCGTGGGTGGGGGTCCATCTTTGGGACCACTGTCGG
Ed_35_3         CCTCGTGGGTGGGGGTCCATCTTTGGGACCACTGTCGG
Ed_36_1         CCTCGTGGGTGGGGGTCCATCTTTGGGACCACTGTCGG

WT (Reference)  --------------------------------------

Ed_23_1         CAGAGGCATCTTGAACGATAGCCTTTCCTTTATCGCAA
Ed_31_1         CAGAGGCATCTTGAACGATAGCCTTTCCTTTATCGCAA
Ed_34_1         CAGAGGCATCTTGAACGATAGCCTTTCCTTTATCGCAA
Ed_35_1         CAGAGGCATCTTGAACGATAGCCTTTCCTTTATCGCAA
Ed_35_2         CAGAGGCATCTTGAACGATAGCCTTTCCTTTATCGCAA
Ed_35_3         CAGAGGCATCTTGAACGATAGCCTTTCCTTTATCGCAA
Ed_36_1         CAGAGGCATCTTGAACGATAGCCTTTCCTTTATCGCAA

WT (Reference)  --------------------------------------

Ed_23_1         TGATGGCATTTGTAGGTGCCACCTTCCTTTTCTACTGT
Ed_31_1         TGATGGCATTTGTAGGTGCCACCTTCCTTTTCTACTGT
Ed_34_1         TGATGGCATTTGTAGGTGCCACCTTCCTTTTCTACTGT
Ed_35_1         TGATGGCATTTGTAGGTGCCACCTTCCTTTTCTACTGT
Ed_35_2         TGATGGCATTTGTAGGTGCCACCTTCCTTTTCTACTGT
Ed_35_3         TGATGGCATTTGTAGGTGCCACCTTCCTTTTCTACTGT
Ed_36_1         TGATGGCATTTGTAGGTGCCACCTTCCTTTTCTACTGT
```

```
WT (Reference)   --------------------------------------

Ed_23_1          CCTTTTGATGAAGTGACAGATAGCTGGGCAATGGAATC
Ed_31_1          CCTTTTGATGAAGTGACAGATAGCTGGGCAATGGAATC
Ed_34_1          CCTTTTGATGAAGTGACAGATAGCTGGGCAATGGAATC
Ed_35_1          CCTTTTGATGAAGTGACAGATAGCTGGGCAATGGAATC
Ed_35_2          CCTTTTGATGAAGTGACAGATAGCTGGGCAATGGAATC
Ed_35_3          CCTTTTGATGAAGTGACAGATAGCTGGGCAATGGAATC
Ed_36_1          CCTTTTGATGAAGTGACAGATAGCTGGGCAATGGAATC

WT (Reference)   --------------------------------------

Ed_23_1          CGAGGAGGTTTCCCGATATTACCCTTTGTTGAAAAGTC
Ed_31_1          CGAGGAGGTTTCCCGATATTACCCTTTGTTGAAAAGTC
Ed_34_1          CGAGGAGGTTTCCCGATATTACCCTTTGTTGAAAAGTC
Ed_35_1          CGAGGAGGTTTCCCGATATTACCCTTTGTTGAAAAGTC
Ed_35_2          CGAGGAGGTTTCCCGATATTACCCTTTGTTGAAAAGTC
Ed_35_3          CGAGGAGGTTTCCCGATATTACCCTTTGTTGAAAAGTC
Ed_36_1          CGAGGAGGTTTCCCGATATTACCCTTTGTTGAAAAGTC

WT (Reference)   --------------------------------------

Ed_23_1          TCAATAGCCCTTTGGTCTTCTGAGACTGTATCTTTGAT
Ed_31_1          TCAATAGCCCTTTGGTCTTCTGAGACTGTATCTTTGAT
Ed_34_1          TCAATAGCCCTTTGGTCTTCTGAGACTGTATCTTTGAT
Ed_35_1          TCAATAGCCCTTTGGTCTTCTGAGACTGTATCTTTGAT
Ed_35_2          TCAATAGCCCTTTGGTCTTCTGAGACTGTATCTTTGAT
Ed_35_3          TCAATAGCCCTTTGGTCTTCTGAGACTGTATCTTTGAT
Ed_36_1          TCAATAGCCCTTTGGTCTTCTGAGACTGTATCTTTGAT

WT (Reference)   --------------------------------------

Ed_23_1          ATTCTTGGAGTAGACGAGAGTGTCGTGCTCCACCATGT
Ed_31_1          ATTCTTGGAGTAGACGAGAGTGTCGTGCTCCACCATGT
Ed_34_1          ATTCTTGGAGTAGACGAGAGTGTCGTGCTCCACCATGT
Ed_35_1          ATTCTTGGAGTAGACGAGAGTGTCGTGCTCCACCATGT
Ed_35_2          ATTCTTGGAGTAGACGAGAGTGTCGTGCTCCACCATGT
Ed_35_3          ATTCTTGGAGTAGACGAGAGTGTCGTGCTCCACCATGT
Ed_36_1          ATTCTTGGAGTAGACGAGAGTGTCGTGCTCCACCATGT

WT (Reference)   --------------TCAAAATTTCTCTCTAGGTTATTT

Ed_23_1          TGGCAAGCTGCTCTT----ATTTCTCTCTAGGTTATTT
Ed_31_1          TGGCAAGCTGCTCTTCAAAATTTCTCTCTAGGCTATTT
Ed_34_1          TGGCAAGCTGCTCTTCAAAATTTCTCTCCAGGTTATTT
Ed_35_1          TGGCAAGCTGCTCTTCAAAATTTCTCTCTAGGTTATTT
Ed_35_2          TGGCAAGCTGCTCTTCAAAATTTCTCTCTAGGTTATTT
Ed_35_3          TGGCAAGCTGCTCTTCAAAATTTCTCTCTAGGTTATTT
Ed_36_1          TGGCAAGCTGCTCTTCAAAATTTCCCTATAGGTTATTT
```

```
WT (Reference)  TTAATCTCAAGATAAAAATGCACTGATCAAGATTGGAA

Ed_23_1         ACAATCTCAAGATAAAAATGCACTGATCAAGATTGGAA
Ed_31_1         TTAATCTCAAGATAAAAATGCACTGATCAAGATTGGAA
Ed_34_1         -TAATCTCAAGATAAAAATGCACTGATCAAGATTGGAA
Ed_35_1         TTAATCTCAAGATAAAAATGCACTGATCAAGATTGGAA
Ed_35_2         TTAATCTCAAGATAAAAATGCACTGATCAAGATTGGAA
Ed_35_3         TTAATCTCAAGATAAAAATGCACTGATCAAGATTGGAA
Ed_36_1         TTAATCTCAAGATAAAAATGCACTGATCAAGATTGGAA

WT (Reference)  TTCTGTTATATATATCATTCAATATTTATAATTTGAAT

Ed_23_1         TTCTGTTATATATATCATTCAATATTTATAATTTGAAT
Ed_31_1         TTCTGTTATATATATCATTCAATATTTAT-----GAAT
Ed_34_1         TTCTGTTATATATATCATTCAATATTTATAATTTGAAT
Ed_35_1         TTCTGTTATATATATCATTCAATATTTATAATTTGAAT
Ed_35_2         TTCTGTTATATATATCATTCAATATTTATAATTTGAAT
Ed_35_3         TTCTGTTATATATATCATTCAATATTTATAATTTGAAT
Ed_36_1         TTCTGTTATATATATCATTCAATATTTATAATTTGAAT

WT (Reference)  TTAAATTCAAAAATAAATTAAATTAAAATTATTTTATT

Ed_23_1         TTAAATTCAAAAATAAATTAAATTAAAATTATTTTATT
Ed_31_1         TTAAATTCAAAAATAAATTAAATTAAAATTATTTTATT
Ed_34_1         TTAAATTCAAAAATAAATTAAATTAAAATTATTTTATT
Ed_35_1         TTAAATTCAAAAATAAATTAAATTAAAATTATTTTATT
Ed_35_2         TTAAATTCAAAAATAAATTAAATTAAAATTATTTTATT
Ed_35_3         TTAAATTCAAAAATAAATTAAATTAAAATTATTTTATT
Ed_36_1         TTAAATTCAAAAATAAATTAAATTAAAATTATTTTATT

WT (Reference)  CAAATTTCAAATACAATATTAA---GTACCTAAACATA

Ed_23_1         CAAATTTCAAATACAATATTAA---GTAC-----CATA
Ed_31_1         CAAATTTCAAATACAATATTAA--CGTACCTAAACATA
Ed_34_1         CAAATTT---ATACAATATTAA---GTACCTAAACATA
Ed_35_1         CAAATTTCAAATACAATATTAA---GTACCTAAACATA
Ed_35_2         CAAATTTCAAATACAATATTAA---GTACCTAAACATA
Ed_35_3         CAAATTTCAAATACAATATTAA---GTACCTAAACATA
Ed_36_1         CAAATTTCAAATACAATATTAA---GTACCTAAACATA

WT (Reference)  ATTTTAAGATTT--AAACATTCATATTTAGTAACTACT

Ed_23_1         ATTTTAAGATTT---AACATTCATATTTAGTAACTACT
Ed_31_1         ATTTTAAGATTT--AAACATTCATATTTAGTAACTACT
Ed_34_1         ATTTTAAGATTT--AAACATTCATATTTAGTAACTACT
Ed_35_1         ATTTTAAGATTT--AAACATTCATATTTAGTAACTACT
Ed_35_2         ATTTTAAGATTT--AAACATTCATATTTAGTAACTACT
Ed_35_3         ATTTTAAGATTT--AAACATTCATATTTAGTAACTACT
Ed_36_1         ATTTTAAGATTT--AAACATTCATATTTAG---CTACT
```

```
WT (Reference)    AGATTATTGACTCG-CGTTACATTGTATATCAAATAAT

Ed_23_1           AGATTATTGACTCG-CGTTACATTGTATATCAAATAAT
Ed_31_1           A----ATTGACTCG-CGTTACATTGTATATCAAATAAT
Ed_34_1           AGATTATTGACTCGTCGTTACATTGTATATCAAATAAT
Ed_35_1           AGATTATTGACTCG-CGTTACATTGTATATCAAATAAT
Ed_35_2           AGATTATTGACTCG-CGTTACATTGTATATCAAATAAT
Ed_35_3           AGATTATTGACTCG-CGTTACATTGTATATCAAATAAT
Ed_36_1           AGATTATTGACTCG-CGTTACATTGTATATCAAATAAT

WT (Reference)    TTTTTTAACTTAAAAAAAAACAAGCAAGTATAGCTAAT

Ed_23_1           TTTTTTAACTTAAAAAAAAACAAGCAAGTATAGCTAAT
Ed_31_1           TTTTTTAACTTAAAAAAAAACAAGCAAGTATAGCTAAT
Ed_34_1           TTTTTTAACTTAAAAAAAAACAAGCAAGTATAGCTAAT
Ed_35_1           TTTTTTAACTTAAAAAAAAACAAGCAAGTATAGCTAAT
Ed_35_2           TTTTTTAACTTAAAAAAAAACAAGCAACTATAGCTAAT
Ed_35_3           TTTTTTAACTTAAAAAAAAACAAGCAAGTATAGCTAAT
Ed_36_1           TTTTTTAACTTAAAAAAAAACAAGCAAGTATAGCTAAT

WT (Reference)    ATTTTTCGAGTAG---AGATATTCTTTTAAATCGTGTC

Ed_23_1           ATTTTTCGAGTAG---AGATATTCTTTTAAATCGTGTC
Ed_31_1           ATTTTTCGAGTAG---AGATATTCTTTTAAATCGTGTC
Ed_34_1           ATTTTTCGAGTAG---AGATATTCTTTTAAATCGTGTC
Ed_35_1           ATTTTTCGAGTAG---AGATATTCTTTTAAATCGTGTC
Ed_35_2           ATTTTTCGAGTAG---AGATATTCTTTTAAATCGTGTC
Ed_35_3           ATTTTTCGAGTAG---AGATATTCTTTTAAATCGTGTC
Ed_36_1           ATTTTTCGAGTAGAGAAGATATTCTTTTAAATCGTGTC

WT (Reference)    AAAGTTGATCCGAT----CAACTCGACGAGTCAAAAAA

Ed_23_1           AAAGTTGATCCGATCAACCAACTCGACGAGTCAAAAAA
Ed_31_1           AAAGTTGATCCGAT----CAACTCGACGAGTCAAAAAA
Ed_34_1           AAAGTTGATCCGAT----CAACTCGACGAGTCAAAAAA
Ed_35_1           AAAGTTGATCCGAT----CAACTCGACGAGTCAAAAAA
Ed_35_2           AAAGTTGATCCGAT----CAACTCGACGAGTCAAAAAA
Ed_35_3           AAAGTTGATCCGAT----CAACTCGACGAGTC--AAAA
Ed_36_1           AAAGTTGATCCGAT----CAACTCGACGAGTCAAAAAA

WT (Reference)    TAACCTGAA-CGATTGAAAAAAAAACATAGTTTGACTT

Ed_23_1           TAACCTGAA-CGATTGAAAAAAAAACATAGTTTGACTT
Ed_31_1           TAACCTGAA-CGATTGAAAAAAAAACATAGTTTGACTT
Ed_34_1           TAACCTGAA-CGATTGAAAAAAAAACATAGTTTGACTT
Ed_35_1           TAACCTGAA-CGATTGAAAAAAAAACATAGTTTGACTT
Ed_35_2           TAACCTGAA-CGATTGAAAAAAAAACATAGTTTGACTT
Ed_35_3           TAACCTGAA-CGATTGAAAAAAAAACATAGTTTGACTT
Ed_36_1           TAACCTGAA-CGATTGAAAAAAAAACATAGTTTGACTT
```

```
WT (Reference)    AAAATAACTCAAGATGACATACTTTTTATAAAATATTA

Ed_23_1           AAAATAACTCAAGATGACATACTTTTTATAAAATATTA
Ed_31_1           AAAATAACTCAAGATGACATACTTTTTATAAAATATTA
Ed_34_1           AAAATAACTCAAGATGACATACTTTTTATAAAATATTA
Ed_35_1           AAAATAACTCAAGATGACATACTTTTTATAAAATATTA
Ed_35_2           AAAATAACTCAAGATGACATACTTTTTATAAA-TATTA
Ed_35_3           AAAATAACTCAAGATGACATACTTTTTATAAAATATTA
Ed_36_1           AAAATAACTCAAGATGACATACTTTTTATAAAATATTA

WT (Reference)    AGACGACAACATATTAGATCGACTCGGGTTAATTCCGG

Ed_23_1           AGACGACAACATATTAGATCGACTCGG----ATTCCGG
Ed_31_1           AGACGACAACATATTAGATCGACTCGGGTTAATTCCGG
Ed_34_1           AGACGACAACATATTAGATCGACTCGGGTTAATTCCGG
Ed_35_1           AGACGACAACATATTAGATCGACTCGGGTTAATTCCGG
Ed_35_2           AGACGACAACATATTAGATCGACTCGGGTTAATTCCGG
Ed_35_3           AGACGACAACATATTAGATCGACTCGGGTTAATTCCGG
Ed_36_1           AGACGACAACATATTAGATCGACTCGGGTTAATTCCGG

WT (Reference)    TTAATCTTTCAAATTCATGTCTCGAGTCATGAAATTAT

Ed_23_1           TTAATCTTTCAAATTCATGTCTCGAGTCAT---ATTAT
Ed_31_1           TTAATCTTTCAAATTCATGTCTCGAGTCATGAAATTAT
Ed_34_1           TTAATCTTTCAAATTCATGTCTCGAGTCATGAAATTAT
Ed_35_1           TTAATCTTTCAAATTCATGTCTCGAGTCATGAAATTAT
Ed_35_2           T-AATCTTTCAAATTCATGTCTCGAGTCATGAAATTAT
Ed_35_3           TTAATCTTTCAAATTCATGTCTCGAGTCATGAAATTAT
Ed_36_1           TTAATCTTTCAAATTCATGTCTCGAGTCATGAAATTAT

WT (Reference)    GATAACCCCATATAAAACAAATCAAAACAAATTCTAAA

Ed_23_1           GATAACCCCATATAAAACAAATCAAAACAAATTCTAAA
Ed_31_1           GATAACCCCATATATTGCAAATCAAAACAAATTCTAAA
Ed_34_1           GATAACCCCATATAAAACAAATCAAAACAAATTCTAAA
Ed_35_1           GATAACCCCATATAAAACAAATCAAAACAAATTCTAAA
Ed_35_2           GATAACCCCATATAAAACAAATCAAAACAAATTCTAAA
Ed_35_3           GATAACCCCATATAAAACAAATCAAAACAAATTCTAAA
Ed_36_1           GATAACCCCATATAAAACAAATCAAAACAAATTCTAAA

WT (Reference)    ATACAATTCTCAATAAACTCATTATTGAATGATGAAAT

Ed_23_1           ATACAATTCTCAATAAACTCATTATTGAATGATGAAAT
Ed_31_1           ATACAATTCTCAATAAACTCATTATTGAATGATGAAAT
Ed_34_1           ATACAA-TCTCAATAAACTCATTATTGAATGATGAAAT
Ed_35_1           ATACAATTCTCAATAAACTCATTATTGAATG--GAAAT
Ed_35_2           ATACAATTCTCAATAAACTCATTATTGAATGATGAAAT
Ed_35_3           ATACAATTCTCAATAAACTCATTATTGAATGATGAAAT
Ed_36_1           ATACAATTCTCAATAAACTCATTATTGAATGATGAAAT
```

```
WT (Reference)   TGAGAAAAAATCAATCTTAAAGAAAGGACACAATAAAA

Ed_23_1          TGAGAAAAAATCAATCTTAAAGAAAGGACACAATAAAA
Ed_31_1          TGAGAAAAAATCAATCTTAAAGAAAGGACACAATAAAA
Ed_34_1          TGAGAAAAAATCAATCTTAAAGAAAGGACACAATAAAA
Ed_35_1          TGAGAAAAAGTCAATCTTAAAGAAAGGACACAATAAAA
Ed_35_2          TGAGAAAAAATCAATCTTAAAGAAAGGACACAAT-AAA
Ed_35_3          TGAGAAAAAATCAATCTTAAAGAAAGGACACAATAAAA
Ed_36_1          TGAGAAAAAATCAATCTTAAAGAAAGGACACAATAAAA

WT (Reference)   CCACCTGAGTCTATTTGAGTTAACTTACAAATCCGTGA

Ed_23_1          CCACCTGAGTCTATTTGAGTTAACTTACAAATCCGTGA
Ed_31_1          CCACCTGAGTCTATTTGAGTTAACTTACAAATCCGTGA
Ed_34_1          CCACCTGAGTCTATTTGAGTTAACTTACAAATCCGTGA
Ed_35_1          CCACCTGAGTCTATTTGAGTTAACTTACAAACACGTGA
Ed_35_2          CCACCTGAGTCTATTTGAGTTAACTTACAAATCCGTGA
Ed_35_3          CCACCTGAGTCTATTTGAGTTAACTTACAAATCCGTGA
Ed_36_1          CCACCTGAGTCTATTTGAGTTAACTTACAAATCCGTGA

WT (Reference)   TTCGGGTCATGA--AACTATGATAACTTTATAAAACAC

Ed_23_1          TTCGGGTCATGA--AACTATGATAACCACATAAAACAC
Ed_31_1          TTCGGGTCATGA--AACTATGATAACTTTATAAAACAC
Ed_34_1          TTCGGGTCATGA--AACTATGATAACTTTATAAAACAC
Ed_35_1          TTCGGGTCATGA--AACTATGATAACTTTATAAAACAC
Ed_35_2          TTCGGGTCATGA--AACTATGATAACTTTATAAAACAC
Ed_35_3          TTCGGGTCTACA--AACTATGATAACTTTATAAAACAC
Ed_36_1          TTCGGGTCAT----AACTATGATAACTTTATAAAACAC

WT (Reference)   AAATCAAAATAAATGACAAAGCTTAATTCATAATCAAC

Ed_23_1          AAATCAAAATAAATGACAAAGCTTAATTCATAATCAAC
Ed_31_1          AAATCAAAATAAATGACAAAGCTTAATTCATAATCAAC
Ed_34_1          AAATCAAAATAAATGACAAAGCTTAATTCATAATCAAC
Ed_35_1          AAATCAAAATAAATGACAAAGCTTAATTCATAATCAAC
Ed_35_2          AAATCAAAATAAATGACAAAGCTTAATTCATAATCAAC
Ed_35_3          AAATCAAAATAAATGACAAAGCTTAATTCATAATCAAC
Ed_36_1          AAATCAAAATAAATGACAAAGCTTAATTCATAATCAAC

WT (Reference)   CAAATATTGAAGATGCTGAATGATGAAACCAAAAAAAA

Ed_23_1          CAAATATTGAAGAT
Ed_31_1          CAAATATTGAAGAT
Ed_34_1          CAAATATTGAAGAT
Ed_35_1          CAAATATTGAAGAT
Ed_35_2          CAAATATTGAAGAT
Ed_35_3          CAAATATTGAAGAT
Ed_36_1          CAAATATTGAAGATGCTGAATGATGAAACCAAAAAAAA
```

WT (Reference)	AATATCAATTAAAAAATAGACAAAAAAAACTAAAGGCA
Ed_23_1	
Ed_31_1	
Ed_34_1	
Ed_35_1	
Ed_35_2	
Ed_35_3	
Ed_36_1	AATATCAATTAAAACATAGACAAAAAAAACT---GGCA

WT (Reference)	ACCAGGTTAACCCGCCAAACCTGCAACATGAGTCATGA
Ed_23_1	
Ed_31_1	
Ed_34_1	
Ed_35_1	
Ed_35_2	
Ed_35_3	
Ed_36_1	ACCAGGTTAACCCGCCAAACCTGCAACATGAGTCATGA

WT (Reference)	GACTGAGATAACAATAAAGAAAGA
Ed_23_1	
Ed_31_1	
Ed_34_1	
Ed_35_1	
Ed_35_2	
Ed_35_3	
Ed_36_1	GACTGAGATAACAATA
